# Supplementary material for: HDAC11 inhibition triggers bimodal thermogenic pathways to circumvent adipocyte catecholamine resistance
Source: J Clin Invest. 2023 Oct 2;133(19):e168192. doi: 10.1172/JCI168192 (PMC10541202; doi:10.1172/JCI168192)

Supplemental Figure 1

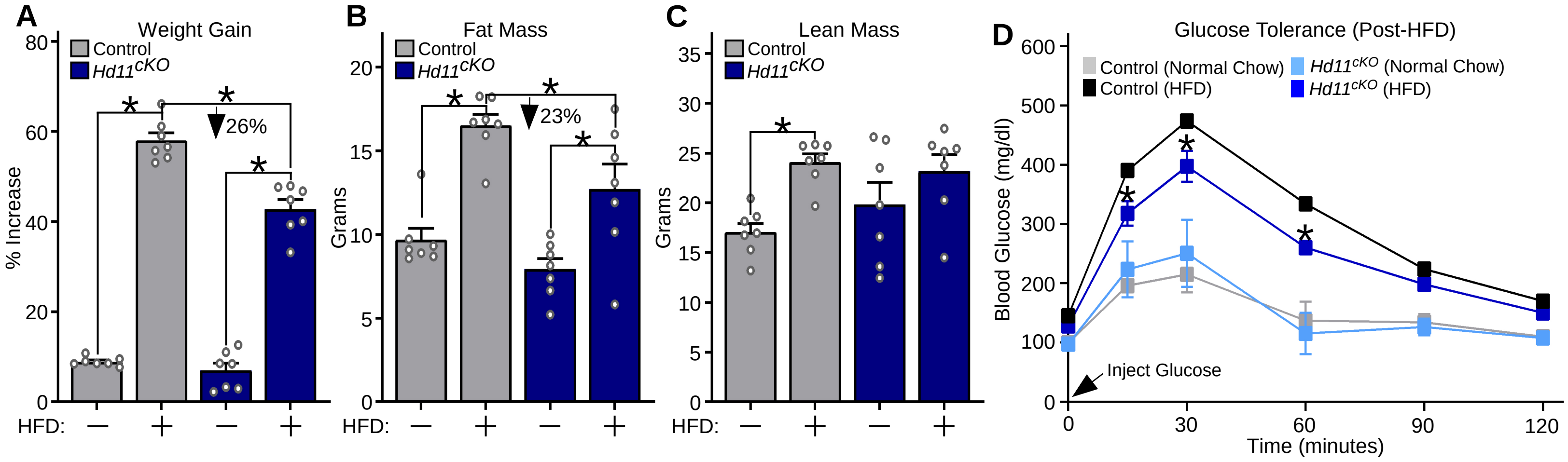

Supplemental Figure 1. Adipocyte-specific deletion of HDAC11 reduces high-fat feeding-induced weight gain. Adipocyte-specific HDAC11 knockout mice (*Hd11*^{cKO}) and *Hdac11*$^{fl/fl}$ controls were fed a high-fat diet (HFD) or normal chow for 16 weeks. (**A**) Percent weight gain over the 16-week period. Quantitative MRI was used to determine fat mass (**B**) and lean mass (**C**) in the mice. Data are presented as mean +SEM, with *P<0.05 vs. as determined by one-way ANOVA with Tukey's multiple comparisons test; each point represents an independent biological replicate. (**D**) Glucose tolerance was assessed prior to animal sacrifice. Data are depicted as mean ±SEM, with *P<0.05 comparing cKO +HFD vs. Control + HFD mice at a given time, as determined by two-way ANOVA with Sidak's multiple comparisons test; n=3 and n=7 biological replicates/group for normal chow and HFD, respectively.

Supplemental Figure 2

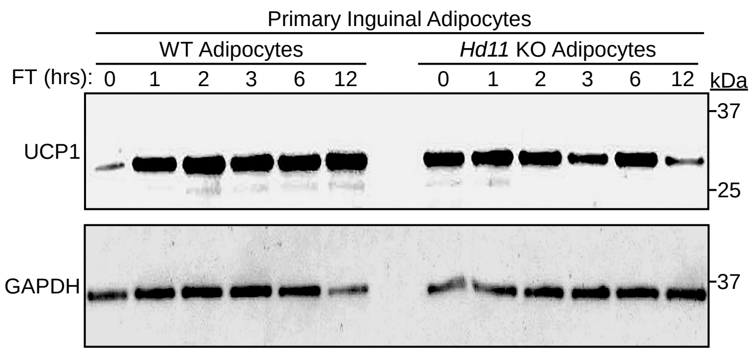

Supplemental Figure 2. Validation of FT895 selectivity for HDAC11. Primary adipocytes were isolated from inguinal white adipose tissue from WT and *Hdac11* global knockout (*Hd11* KO) mice and were exposed, in culture, to FT895 for the indicated times prior to homogenization and immunoblotting to assess UCP1 expression; GAPDH served as a loading control.

Supplemental Figure 3

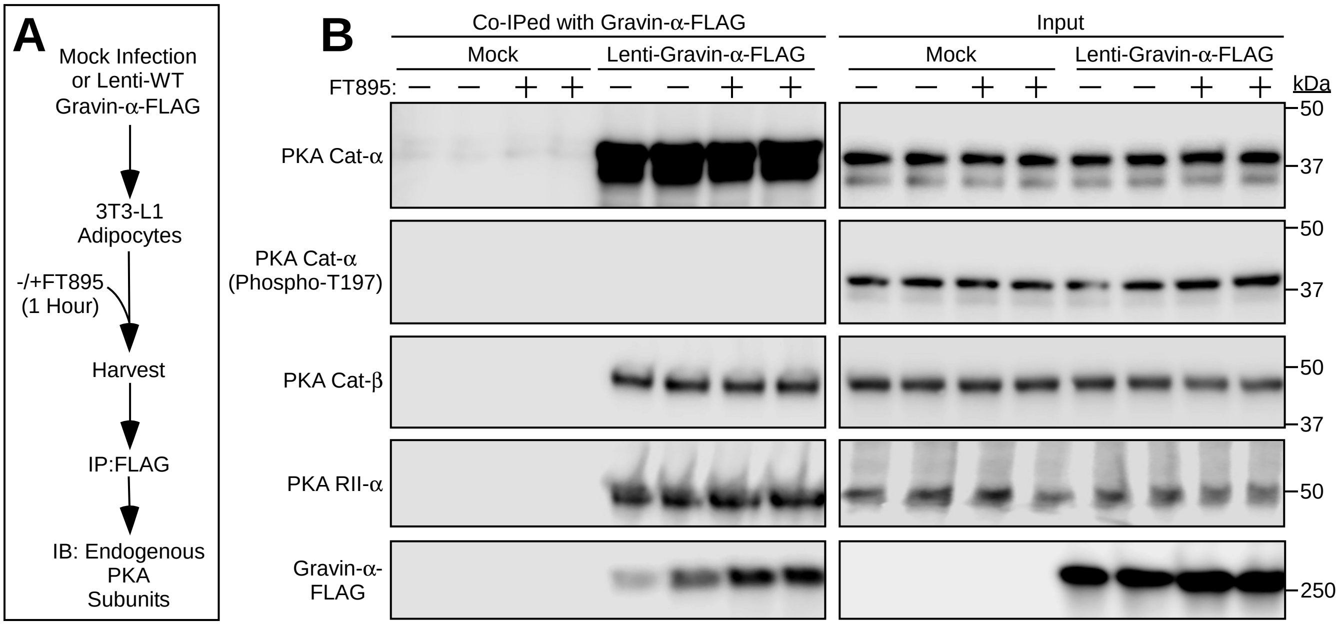

Supplemental Figure 3. HDAC11 inhibition does not alter PKA subunit binding to gravin-α in adipocytes. (**A**) Schematic representation of the co-immunoprecipitation (co-IP) experiment with 3T3-L1 protein homogenates. (**B**) Treatment with the HDAC11 inhibitor, FT895, did not alter association of the indicated endogenous PKA subunits with FLAG-tagged gravin-α.

Supplemental Figure 4

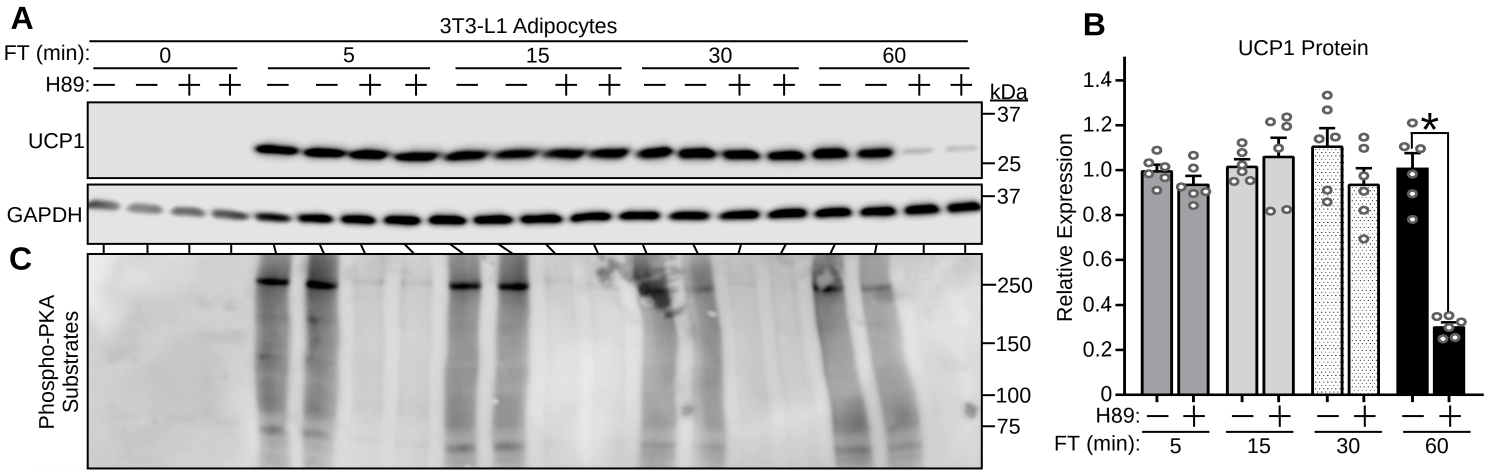

Supplemental Figure 4. Kinetics of PKA-independent and PKA-dependent induction of UCP1 expression upon HDAC11 inhibition in adipocytes. (**A**) 3T3-L1 adipocytes were pretreated with the PKA inhibitor, H89, for 30 minutes prior to exposure to FT895 for the indicates times. Immunoblotting was performed to assessed the indicated proteins. (**B**) Densitometric analysis of UCP1 expression in (**A**), as well as from two additional independent experiments (blots not shown; 2 technical replicates each from 3 experiments), normalized to GAPDH and plotted as fold-change relative to FT895 (FT) treatment without H89. Data are presented as mean +SEM, with *P<0.05 as determined by one-way ANOVA with Tukey's multiple comparisons test. (**C**) Immunoblotting for phosphorylation of PKA substrates.

Supplemental Figure 5

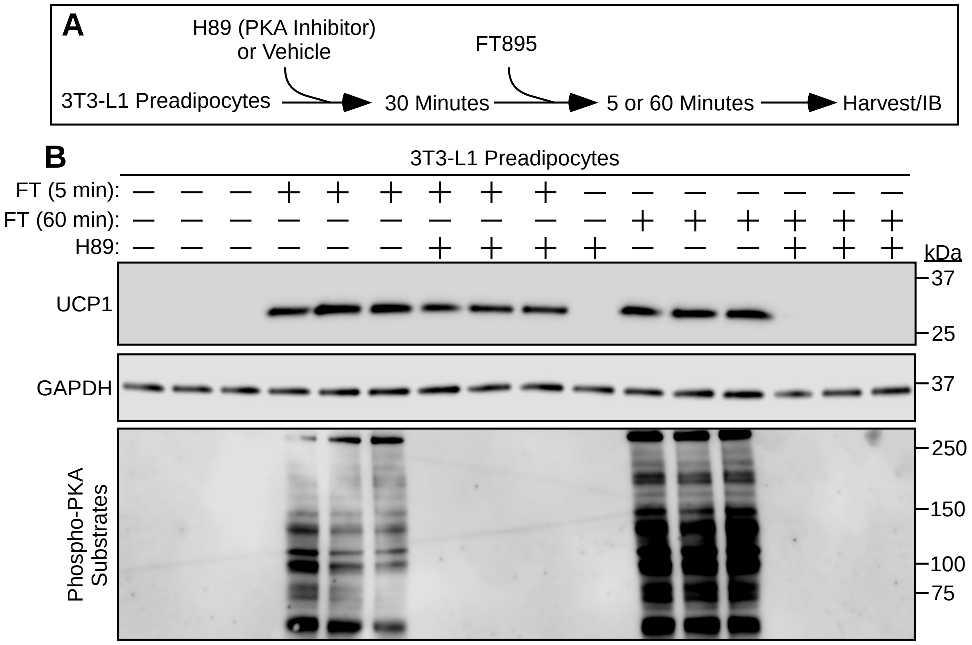

Supplemental Figure 5. HDAC11 inhibition triggers PKA-independent and PKA-dependent induction of UCP1 protein expression in preadipocytes. (**A**) Schematic depiction of the experiment employing undifferentiated 3T3-L1 preadipocytes. (**B**) Immunoblot (IB) assessment of the indicated proteins.

Supplemental Figure 6

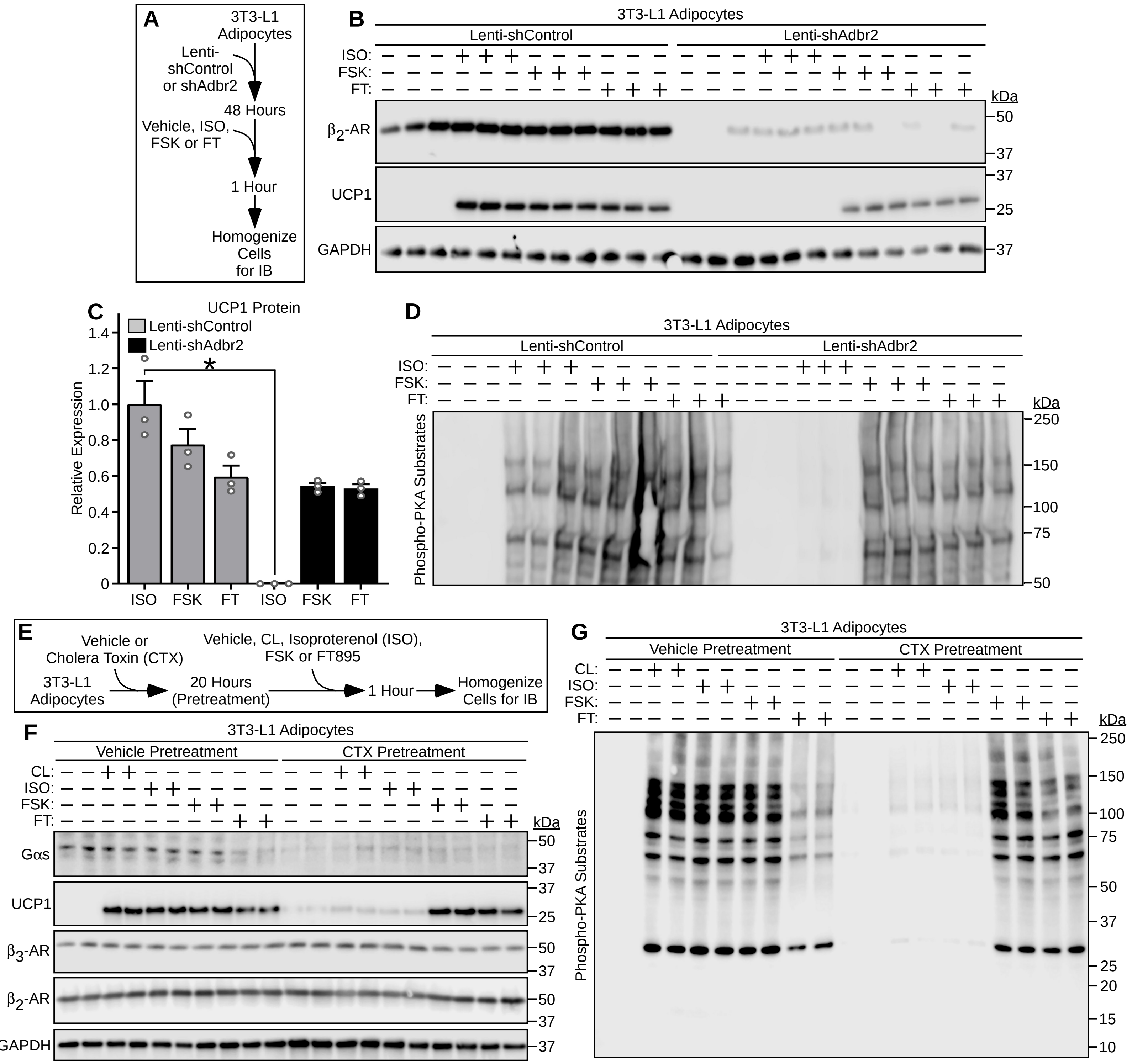

Supplemental Figure 7

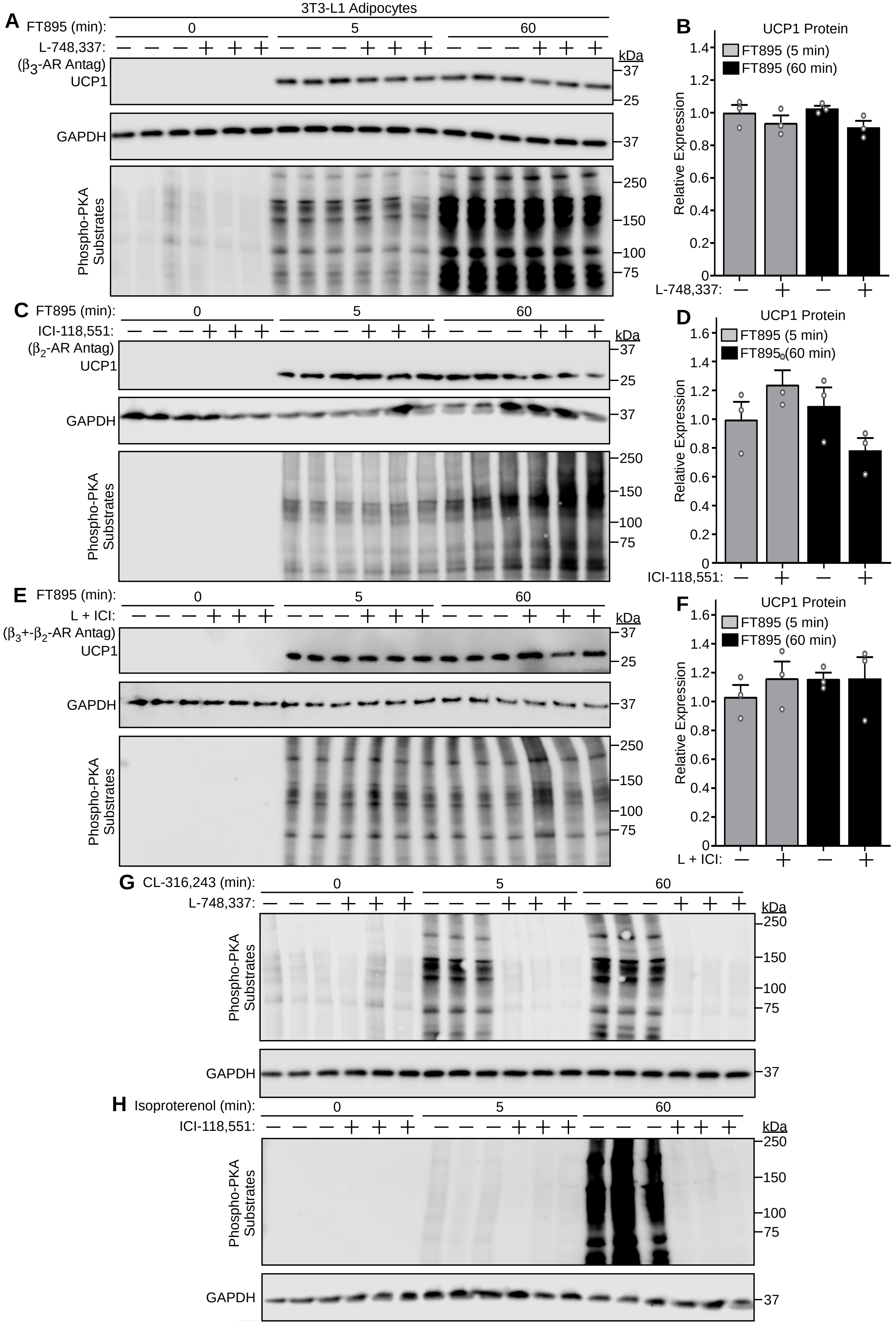

Supplemental Figure 8

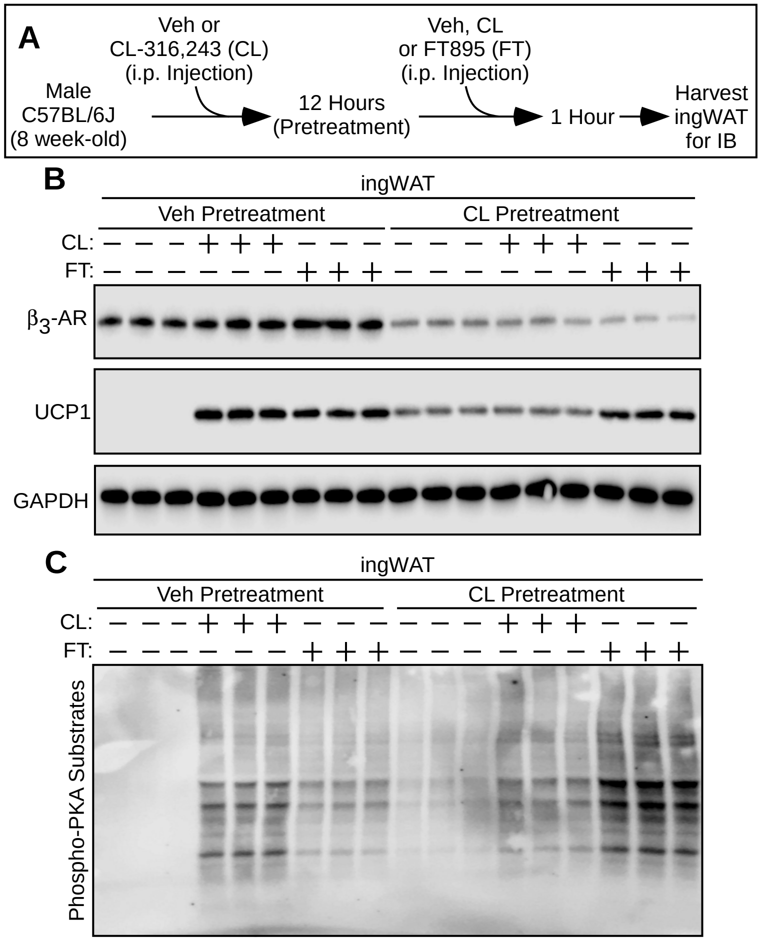

Supplemental Figure 8. HDAC11 inhibition circumvents catecholamine resistance in mouse ingWAT *in vivo*. (**A**) Schematic representation of the *in vivo* experiment to determine if HDAC11 inhibition promotes UCP1 expression and PKA signaling in the context of downregulated β3-adrenergic receptor (β3-AR) expression in inguinal white adipose tissue (ingWAT). (**B**) Immunoblot analysis of the indicated proteins in homogenates of ingWAT in mice pretreated with vehicle (Veh) or CL-316,243 (CL) for 12 hours followed by 1-hour treatment with vehicle (-), CL or FT895 (FT); n=3 biological replicates/condition. (**C**) These same samples were independently immunoblotted with an antibody that recognizes proteins containing phospho-serine/threonine residues within a consensus PKA target site (RRXS*/T*).

Supplemental Figure 9

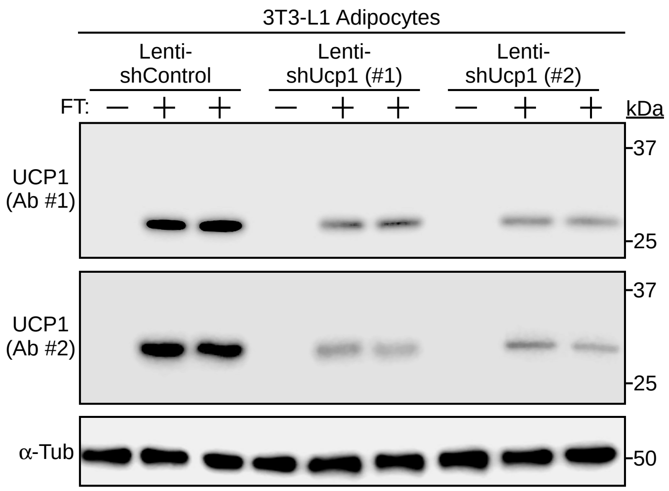

Supplemental Figure 9. Validation of UCP1 antibody specificity. 3T3-L1 adipocytes were infected with lentivirus encoding a control short-hairpin RNA (Lenti-shControl) or viruses encoding two independent shRNAs to target *Ucp1* mRNA (shUcp1). Cells were homogenized 24-hours after infection and immunoblot analysis was performed to assess UCP1 protein expression; α-tubulin (α-Tub) served as a loading control.

Supplemental Table 1. Primer sequences for genotyping and qRT-PCR

Primer Name	Application	Species	Primer Sequence
Ucp1 F	RT-qPCR	Human	5'-AGTTCCTCACCGCAGGGAAAGA-3'
Ucp1 R	RT-qPCR	Human	5'-GTAGCGAGGTTTGATTCCGTGG-3'
Ucp1 F	RT-qPCR	Mouse	5'-CCGAAACTGTACAGCGGTCT-3'
Ucp1 R	RT-qPCR	Mouse	5'-CCGAGAGAGGCAGGTGTTTC-3'
18S F	RT-qPCR	Human, Mouse	5'-GCCGCTAGAGGTGAAATTCTTA-3'
18S R	RT-qPCR	Human, Mouse	5'-CTTTCGCTCTGGTCCGTCTT-3'
Hdac11 flox F	DNA PCR	Mouse	5'-GTGCAGGCCTTGGGCCTTGGCA-3'
Hdac11 flox R	DNA PCR	Mouse	5'-CTGAGGAGGTAGTATGGATAG-3'

Supplemental Table 2. Chemicals and reagents

Item	Vendor	Catalog #
Dulbecco's Modification of Eagle's Medium (DMEM)	Corning	10-013-CV
(Minimum Essential Medium α (MEMα)	Corning	10-022-CV
Fetal Bovine Serum, HyClone Characterized FBS, US Origin	GE Healthcare	SH30071.02
Newborn Calf Serum, heat inactivated, New Zealand origin	Thermo Scientific	26010074
Penicillin-Streptomycin-L-Glutamine, 100X	Corning	30-009-CI
Insulin-Transferrin-Selenium, 100X	Thermo Gibco	41400045
Dexamethasone	Sigma Aldrich	D4902
3-Isobutyl-1-methylxanthine, IBMX	Sigma Aldrich	I5879
Rosiglitazone	Cayman Chemicals	71740
Collagenase II	Worthington	LS004176
Cell-permeable Cre recombinase, TAT-Cre (Tat-NLS-Cre, HTNC, HTNCre)	Excellgen	EG-1001
Halt™ Protease and Phosphatase Inhibitor Cocktail	Thermo Scientific	78440
Pierce™ BCA Protein Assay Kit	Thermo Scientific	PI23227
4-15% Criterion TGX Precast Midi Protein Gel, 26 well,	BIO-RAD	5671085
4-15% Criterion TGX Precast Midi Protein Gel, 18 well	BIO-RAD	5671084
Nitrocellulose membrane 0.45 µm	BIO-RAD	1620115
Precision Plus Protein Dual Color Standards	BIO-RAD	1610394
Bovine Serum Albumin Fraction V for immunoblotting	Akron Biotech	AAJ64655
Bovine Serum Albumin, fatty acid free for immunofluorescence	Thermo Scientific	A8806
Stainless steel beads 1.6 mm	Next Advance	#SSB16
Alkynyl Myristic Acid (Alk-12)	Click Chemistry Tools	1164

Click-iT® Protein Reaction Buffer Kit	Thermo Scientific	C10276
Biotin Azide (PEG4 carboxamide-6-Azidohexanyl Biotin)	Thermo Scientific	B10184
Pierce™ Streptavidin Magnetic Beads	Thermo Scientific	88816
FT895; FT	MedChemExpress	2225728-57-2
CL-316,243; CL	Sigma Aldrich	C5976
Isoproterenol; ISO	Calbiochem	420355
Forskolin; FSK	Sigma Aldrich	F6886
L-748,337	Tocris Bioscience	2760
ICI-118,551	Sigma Aldrich	I127
H89	Tocris Bioscience	2910
DMSO	Sigma Aldrich	D8418
N,N-Dimethylacetamide (DMA)	Sigma Aldrich	D137510
Tween-80	Sigma Aldrich	P1754
4,4-Difluoro-1,3,5,7,8-Pentamethyl-4-Bora-3a,4a-Diaza-s-Indacene; BODIPY	Thermo Scientific	D3922
4',6-diamidino-2-phenylindole; DAPI	Thermo Scientific	D1306
D-Biotin	Sigma Aldrich	B4639
D-Pantothenic acid hemicalcium salt	Sigma Aldrich	P5155
3,3′,5-Triiodo-L-thyronine sodium salt; T3	Sigma Aldrich	T6397
Transferrin, human	Sigma Aldrich	T8158
Insulin solution, human	Sigma Aldrich	I9278
Tween-20	Sigma Aldrich	P1754
Glass coverslips, 25 mm	Chemglass	CLS-1763-025
Microscope slides, Diamond White Glass, 25 x 75mm, Charged, 90° Ground Edges, White Frosted	Global Scientific	1358W
Nunc™ Lab-Tek™ II Chamber Slide™	Thermo Scientific	12-565-7
Formaldehyde,16 % methanol-free Ultra Pure	Thermo Scientific	18814-10
VECTASHIELD® HardSet™ Antifade Mounting Medium for immunofluorescence	Vector Labs	H-1400-10
Antigen Unmasking Solution, Citrate-Based	Vector Labs	H-3300-250
BLOXALL® Endogenous Blocking Solution, Peroxidase and Alkaline Phosphatase	Vector Labs	SP-6000-100
ECTASTAIN® Elite® ABC-HRP Kit, Peroxidase (Standard)	Vector Labs	PK-6100
Vector® NovaRED™ Substrate Kit, Peroxidase (HRP)	Vector Labs	SK-4800
Fisher Chemical™ Permount™ Mounting Medium for IHC	Thermo Scientific	SP15-100
Agarose	Life Science Products	A-1705
1kb DNA ladder	Promega	PR-G5711
Protein ladder, Precision Plus Protein™ Dual Color Standards	BIO-RAD	1610394

Non-fat milk powder, Kroger® Instant Non-Fat Dry Milk	Kroger®	0001111083297
PowerUp SYBR Green Master Mix	Thermo Scientific	4368577
TRIzol™ Reagent	Thermo Scientific, Ambion	15596026
Verso cDNA Synthesis Kit	Thermo Scientific	AB1453B
Polybrene	Sigma Aldrich	TR-1003
Polyethylenimine, Linear, PEI	Polysciences Inc.	23966-1

Supplemental Table 3. Patient information for human VAT samples

Age (yrs)	Sex	Ethnicity	BMI Consent	BMI Surgery
30	F	Hispanic	42.0	42.0085992
39	M	Non-Hispanic, Indian	36.9839009	36.9839009
22	F	White	42.0	41.8102305

Supplemental Table 4. Patient information for human SC adipocyte isolation

Age (yrs)	Sex	BMI
29	M	21.1

Supplemental Table 5. Antibodies

Antigen	Vendor	Host	Catalog #
UCP1 #1	Abcam	Rabbit	ab209483
UCP1 #2	Cell Signaling Technology	Rabbit	14670S
GAPDH	Thermo Scientific	Mouse	AM4300
Phospho-PKA substrates	Cell Signaling Technology	Rabbit	9624
α-Tubulin-HRP	Santa Cruz Biotechnology	Mouse	sc-23948
Gravin-α	Proteintech	Rabbit	25199-1-AP
Anti-FLAG-HRP	Sigma Aldrich	Mouse	F1804
Anti-FLAG Agarose Beads	Pierce Thermo Scientific	Rat	A36797
β_3-AR	Abcam	Rabbit	ab94506
β_2-AR	Proteintech	Rabbit	13096-1-AP
HDAC11	From Dr. Edward Seto, George Washington University	Rabbit	
PKA Cat-α	Santa Cruz	Rabbit	SC-903
PKA Cat-α (Phospho-T197)	Cell Signaling Technology	Rabbit	4781
PKA Cat-β	Proteintech	Rabbit	10142-2-AP
PKA RII-α	BD Biosciences	Mouse	612242

anti-Rabbit Alexa Fluor 594 for Immunofluorescence	Thermo Scientific	Goat	A-11012
Anti-Rabbit IgG-HRP	Southern Biotech	Goat	OB4050-05
Anti-Mouse IgG-HRP	Southern Biotech	Goat	OB1031-05
Anti-Rabbit IgG Antibody (H+L), Biotinylated for IHC	Vector Labs	Goat	BA-1000-1.5

Supplemental Material

Expanded Materials and Methods

Adipocyte culture. The 3T3L1 CL-173™ preadipocyte cell line was purchased from ATCC. Cells were maintained and differentiated into mature adipocytes *in vitro* as previously described (3). Undifferentiated cells were seeded on culture dishes and grown to 80-90 % confluency in DMEM supplemented with 10% newborn calf serum and 1X Penicillin-Streptomycin-L-Glutamine. Differentiation of 3T3-L1 cells was induced using DMEM supplemented with 10% fetal bovine serum, 1 μM dexamethasone, 0.5 mM 3-isobutyl-1-methylxanthine, 1 μg/mL insulin for 72 hr. Following this incubation, media was changed to adipocyte maintenance media (DMEM with 1μg/mL insulin, 10% FBS) and replenished every 48h for 8 days or until lipid droplet coverage is 80 – 90 %, as assessed by brightfield microscopy. At this time, 3T3-L1 adipocytes were treated as per the experiments described.

HIB1B brown-like preadipocytes were cultured in DMEM supplemented with 10% FBS and 1X Penicillin-Streptomycin-L-Glutamine. HIB1B adipocytes were differentiated into mature adipocytes at 70% confluency using rosiglitazone (1 μM) for four days. Differentiation was monitored by assessing lipid droplet coverage by brightfield microscopy, as well as high basal UCP1 expression. HIB1B cells were provided by Sheila Collins, Vanderbilt School of Medicine, Nashville, TN, USA.

Mouse primary preadipocytes were isolated from SVF of murine inguinal AT of 10 – 12-week-old male WT, *Hdac11*$^{fl/fl}$ or *Hdac11*^{KO} C57BL/6J mice, as previously described

(3). ingWAT was dissected and washed in pre-warmed DMEM before being transferred to a petri dish containing 10 mL of 0.2% (w/v) collagenase II. The samples were subsequently cut into ~1 mm pieces and further mincing was performed to allow opening up of the tissue using spring scissors (Fine Science Tools). The minced tissue in digestion buffer was then transferred to a 15 mL Falcon tube with the lid loosely fastened and incubated in a shaker at 37 ºC at 400 rpm for 20 minutes, with vortexing every 5 minutes. At the end of the digestion, the tissue slurry was passed through a 70 µm cell strainer into a 50 mL Falcon tube, followed by 30 mL preadipocyte media (5% NBCS, 5% FBS,1% PSG) to stop enzymatic activity. After a brief (2-3 minute) centrifugation at 200g at room temperature, the upper floating mature adipocyte-containing layer was discarded. The cell suspension was then centrifuged at 2000 x g for 5 minutes at room temperature to pellet the SVF-derived preadipocytes. The supernatant was removed and the remaining cell pellet resuspended in pre-warmed preadipocyte media. Cells were plated directly onto 6- well cell culture plates (VWR). Once 90 - 100 % preadipocyte confluency was reached, differentiation was induced by treatment with differentiation induction medium (DMEM, 10% FBS, 0.5% PSG, 1 µg/mL insulin, 0.5 µM dexamethasone, 0.25 mM 3-isobutyl-1- IBMX and 1 um rosiglitazone) for 72 hours. On day 3, induction medium was replaced with maintenance media (DMEM, 10 % FBS, 0.5% PSG, 1 ug/mL insulin). Maintenance media was replenished every 48 hours and maturation followed by observing lipid droplet coverage by brightfield microscopy, with 80-90 % lipid droplet coverage considered fully differentiated. For treatment of cultured adipocytes, the following compounds/concentrations were used: FT895 (10 µM), CL-316,243 (1 µM),

isoproterenol (1 μM), L-748,337 (10 μM), ICI-118,551 (1 μM), forskolin (10 μM), H89 (10 μM), cholera toxin (100 ng/mL) or vehicle control (DMSO; 0.1% final concentration).

Human visceral adipose tissue (VAT) explant acquisition and treatment. Human VAT samples were acquired from bariatric surgery patients under protocols approved by Colorado Multiple Institutional Review Board. All VAT samples were deidentified prior to use in experiments. Patient characteristics are described in Supplemental Table 3. VAT explants were washed, trimmed and rinsed in pre-warmed DMEM and cut with fine scissors into 1 mm x 1 mm pieces for culture in ThinCert™ Tissue Culture Inserts (Greiner Bio-One) submerged in 1 mL DMEM in 6-well tissue culture plates. Human VAT explants were treated with FT895 (100 μM), CL-316,243 (10 μM), isoproterenol (100 μM) or forskolin (100 μM) or vehicle control (DMSO; 0.1% final concentration). VAT explants were cultured and maintained in a humidified cell culture incubator maintained at 37°C with 5% CO_2.

Human subcutaneous (SC) preadipocyte acquisition, culture and differentiation. Human SC adipose tissue samples were acquired from laparoscopic surgeries from volunteers, as described previously, described under protocols approved by Colorado Multiple Institutional Review Board (NCT02654925) (1). Subject information can be found in Supplemental Table 4. Stromal vascular fraction (SVF)-derived preadipocytes were isolated according to published procedures and plated onto 6-well tissue culture dishes in growth media (MEMα, 10% FBS, 0.1X Penicillin-Streptomycin-Glutamine) (2). Once SVF-derived preadipocytes reached 90% confluency, medium was changed to complete

differentiation induction media for up to seven days, with medium refreshed every 48 hours (DMEM, 1X PSG, 33 μM d-Biotin, 17 μM pantothenate, 100 nM dexamethasone, 1μM rosiglitazone, 0.5 mM IBMX, 2nM T3, 10 μg/mL transferrin, 100 nM insulin). Following seven days, complete differentiation medium was switched to maintenance media (DMEM, 1X PSG, 33 μM d-Biotin, 17 μM pantothenate, 10 nM dexamethasone, 100 nM insulin). Differentiation was assessed by lipid droplet coverage by brightfield microscopy (EVOS FL, Life Technologies), with 80-90% lipid droplet coverage considered fully differentiated.

Plasmids and lentivirus generation. p3XFLAG-CMV-14 expression vector encoding full length rat gravin-α was a gift from Dr. Joseph Miano, Augusta University, U.S.A (4). This base vector was used to generate the K1502/K1505 mutant construct of gravin-α, as previously described (3). These constructs were sub-cloned into pLenti CMV Hygro DEST (Addgene #17454). To create short hairpin RNA encoding lentivirus, pLKO.1 plasmids (sequences obtained from the Sigma MISSION™ shRNA library) encoding shRNA for mouse *gravin-α* (TRCN0000088805)*, Ucp1* (TRCN0000114241 and TRCN0000114245), *Adrb3* (TRCN0000027337), *Adrb2* (TRCN0000027549) and a scrambled shRNA control (SHC002) were obtained through the Functional Genomics Facility at the University of Colorado Cancer Center. Lentiviruses were generated by co-transfection of shRNA in pLKO.1 or pLenti overexpression plasmids, psPAX2 and pMD2.G into 293T cells (ATCC #CRL-3216™). The viral supernatant was collected 60 hours after transfection, filtered through a 0.45 μm syringe filter. Viral supernatant was supplemented with polybrene at 1 μl/mL (stock concentration 10mg/mL) and used to directly infect adipocytes. After 24 or

48 hours of infection, cells were used for experiments as indicated. psPAX2 and pMD2.G were gifts from Didier Trono (Addgene plasmids # 12260, #12259).

Assessment of gravin-α myristoylation. Detection of lysine myristoyation on gravin-α was performed using a Click chemistry approach as previously described (3). Cells were supplemented with 50 μM alkynyl myristic acid for 4 hours prior to harvest, then lysed in 1% SDS lysis buffer (50 mM Tris-HCl pH 8.0, 1% (w/v) SDS) with protease and phosphatase inhibitors. Cell lysates were prepared as standard and protein concentration was determined using a BCA Protein Assay Kit. Click chemistry reactions with biotin-azide (50 μM final concentration) were performed at room temperature for 30 minutes with end-over-end rotation with up to 200 μg of protein per sample, and the reactions were terminated by bringing the volume of the sample to 60 μl with 1% SDS lysis buffer. To precipitate and concentrate proteins, 600 μL of methanol, 150 μL of chloroform and 400 μL of 18 MΩ water were added to each sample. Samples were vortexed and centrifuged at 13,000 x g for 5 minutes at room temperature. The upper aqueous supernatant was gently removed and the interface layer containing the protein precipitate was left intact. 450 μL of methanol was added to the tubes, vortexed, and the samples spun at 13,000 x g for 5 minutes to pellet the protein. The methanol supernatant was removed, and the protein pellets were washed once again with 450 μL of methanol or until the protein pellets are white in color. The protein pellets were then air-dried for 15-30 minutes and resolubilized in 1% SDS buffer. Protein concentration was assessed by BCA assay, and 100 μg of biotin-labeled protein was then incubated with 50 μL of streptavidin magnetic beads overnight at 4°C with end-over-end rotation. Following

incubation, beads were collected with a magnetic rack and rinsed three times with washing buffer (Tris-buffered saline containing 0.1% Tween™-20). After the final wash, the supernatant was discarded and 50 uL 2X sample buffer (100 mM Tris pH 6.8, 4% SDS w/v, 20% glycerol v/v, 10% β-mercaptoethanol and bromophenol blue) was added to the beads and boiled at 95 °C for 10 minutes. The samples were further analyzed via immunoblotting using antibody specific for gravin-α, and total gravin-α amount was used for reference input.

Immunoblotting and Immunoprecipitation. Cultured cells were washed with PBS and lysed with RIPA buffer (50mM Tris.HCl pH 8.0, 150mM NaCl, 1% NP-40, 0.5% sodium deoxycholate and 0.1% SDS supplemented with protease and phosphatase inhibitors) for 30 minutes on ice followed by scraping and centrifugation at 13,000 xg for 5 minutes. For AT, RIPA buffer was added to 25-50 mg of tissue in an Eppendorf tube and the tissue was physically disrupted with sterile stainless-steel beads in a bullet blender (Next Advance Bullet Blender 24) for 5 minutes at setting #9 at 4 ºC. Protein concentrations were measured using a BCA assay, and 10 – 30 μg of protein was resolved through 4-15 % pre-cast polyacrylamide gels and transferred onto 0.45 μm nitrocellulose membranes. Membranes were blocked in 5% non-fat milk powder in TBS-Tween (20 mM Tris, 150 mM NaCl, 0.1% Tween 20) and proteins were analysed by immunoblotting using antibodies against specific antigens (Supplemental Table 5). Membranes were incubated with primary antibodies at a final concentration of 1:1000 in 2.5% bovine serum albumin in TBS-Tween for 2 hours at room temperature or overnight at 4 ºC on a rocker. Washes were performed thrice for 10 minutes in TBS-Tween followed by secondary antibody

incubation with horse radish peroxidase (HRP)-conjugated anti-rabbit or anti-mouse secondary antibodies at a final concentration of 1:2000 in 5% non-fat milk in TBS-Tween. Final washes were performed in 1 X TBS-Tween, thrice for 10 minutes, prior to imaging. Immunoblot images were acquired on an Odyssey® XF digital imaging system (LI-COR) using the chemiluminescence program and LI-COR Acquisition software or on a ChemiDoc™ Imaging system (BIO-RAD). Protein abundance was quantified using the LI-COR Image Studio™ Lite software and normalized to respective reference proteins. Immunoblotting with homogenates of 3T3-L1 cells that were infected with two different lentiviral constructs expressing short hairpin RNA sequences against *Ucp1* confirmed the specificity of the anti-UCP1 antibodies that were employed (Supplemental Figure 9).

For co-immunoprecipitation (co-IP) studies, 3T3-L1 adipocytes were infected with lentivirus encoding FLAG-tagged rat gravin-α using 10 ng/mL polybrene or were mock infected (10 ng/mL polybrene only)) and 48 hours post-infection, cells were treated with FT895 (10 μM) or vehicle (DMSO) for 1 hour as indicated. Whole-cell lysates were prepared with mild IP lysis buffer (300 mM NaCl, 0.5% Triton X-100, PBS pH 7.4 with 1 X Halt Protease & Phosphatase Inhibitor Cocktail (Thermo Scientific)) and syringe homogenization with a 25-gauge needle. Whole-cell lysates were collected following centrifugation of lysates at 12000g at 4°C for 10 minutes, and the protein concentrations were determined using BCA assay. For each IP 0.5 mgs of protein were subjected to IP with anti-FLAG (DYKDDDDK) agarose beads (25 µl in 1 ml total volume) for 4 hours at room temperature with end-over-end rotation. Beads were washed three times with mild IP lysis buffer and boiled in sample buffer prior to SDS-PAGE. Whole-cell lysates were used as input controls.

Reverse transcription Quantitative PCR (RT-qPCR) gene expression analysis. RNA was isolated from cells or tissue using TRIzol lysis reagent and 0.5 – 1.0 μg of RNA was reverse transcribed and cDNA synthesized with random hexamers for single strand synthesis. RT-qPCR was performed on a StepOnePlus Real-Time PCR System (Applied Biosystems) using SYBR Green Master Mix and gene-specific primers at a final concentration of 500 nM each for forward and reverse primers. RT-qPCR primer sequences are provided in Table 1. Relative gene expression was calculated using the $2^{-ddCt\Delta CT}$ method and normalized to 18S RNA (5).

Immunofluorescence microscopy. 3T3L1 cells were grown and differentiated on glass coverslips (25 mm diameter) in 6-well tissue culture plates. Cells were fixed with 4% formaldehyde in PBS for at least 20 minutes at room temperature followed by permeabilization in 0.25% Triton X-100 in PBS for 15 minutes. Non-specific antigen binding was blocked by incubating the cells with 5 % bovine serum albumin in PBS for 1 hour at room temperature. Immunostaining was performed by incubating UCP1 primary antibody (Abcam, ab209483) at a concentration of 1:250 in 2.5% BSA in PBS overnight at 4 ºC on a rocker. Cells were washed thrice with PBS and incubated with fluorescent secondary antibody (1:500) along with BODIPY (5 mM) and 4',6-diamidino-2-phenylindole (DAPI) at a final concentration of 300 nM in PBS for one hour at room temperature, rocking gently. Cells were then washed twice in PBS then mounted onto white frosted microscope slides. Imaging was performed on a Zeiss LSM780 confocal microscope (Advanced Light Microscopy Core, University of Colorado Anschutz Medical

Campus) using a 100x objective lens, and images processed through ZEN Black software (Zeiss).

For wholemount tissue imaging, AT was fixed in 4% formaldehyde in PBS overnight at 4 ºC with end-over-end rotation followed by permeabilization in 0.5% Triton X-100 in PBS for 1 hour with end-over-end rotation. Lipid droplets were stained with BODIPY (5 μM) in PBS overnight at 4 ºC with end-over-end rotation. Stained AT was rinsed twice in PBS, mounted in chamber slides and whole mount imaging was performed on a Zeiss LSM780 confocal microscope (Advanced Light Microscopy Core, University of Colorado Anschutz Medical Campus) using a 10x objective lens and processed through ZEN Black software (Zeiss).

UCP1 immunohistochemistry. Epididymal AT from chow fed control (*Hdac11*$^{fl/fl}$) and *Hdac11*^{cKO} was formalin fixed and embedded in paraffin. Tissue sections (5 μm thick) of eWAT were deparaffinized, hydrated, and subjected to antigen retrieval using citrate buffer. Endogenous peroxidase activity was inhibited using blocking for 10 minutes, and sections were subsequently incubated in 5% BSA for 1 hour at room temperature. Tissue sections were incubated overnight with primary anti-UCP1 antibody (Abcam, ab209483) at a final concentration of 1:100, followed by incubation with biotinylated secondary antibody at a final concentration of 1:500, and immunoperoxidase detection was performed. Images were acquired using an Olympus microscope (BX51) using a 10x objective lens equipped with a multicolor camera (DP72).

Supplemental References

1. Gavin KM, Gutman JA, Kohrt WM, Wei Q, Shea KL, Miller HL, et al. De novo generation of adipocytes from circulating progenitor cells in mouse and human adipose tissue. *FASEB J.* 2016;30(3):1096-108.
2. Lee MJ, and Fried SK. Optimal protocol for the differentiation and metabolic analysis of human adipose stromal cells. *Methods Enzymol.* 2014;538:49-65.
3. Bagchi RA, Robinson EL, Hu T, Cao J, Hong JY, Tharp CA, et al. Reversible lysine fatty acylation of an anchoring protein mediates adipocyte adrenergic signaling. *Proc Natl Acad Sci U S A.* 2022;119(7).
4. Streb JW, Kitchen CM, Gelman IH, and Miano JM. Multiple promoters direct expression of three AKAP12 isoforms with distinct subcellular and tissue distribution profiles. *J Biol Chem.* 2004;279(53):56014-23.
5. Livak KJ, and Schmittgen TD. Analysis of relative gene expression data using real-time quantitative PCR and the 2(-Delta Delta C(T)) Method. *Methods.* 2001;25(4):402-8.

Fig. 1E

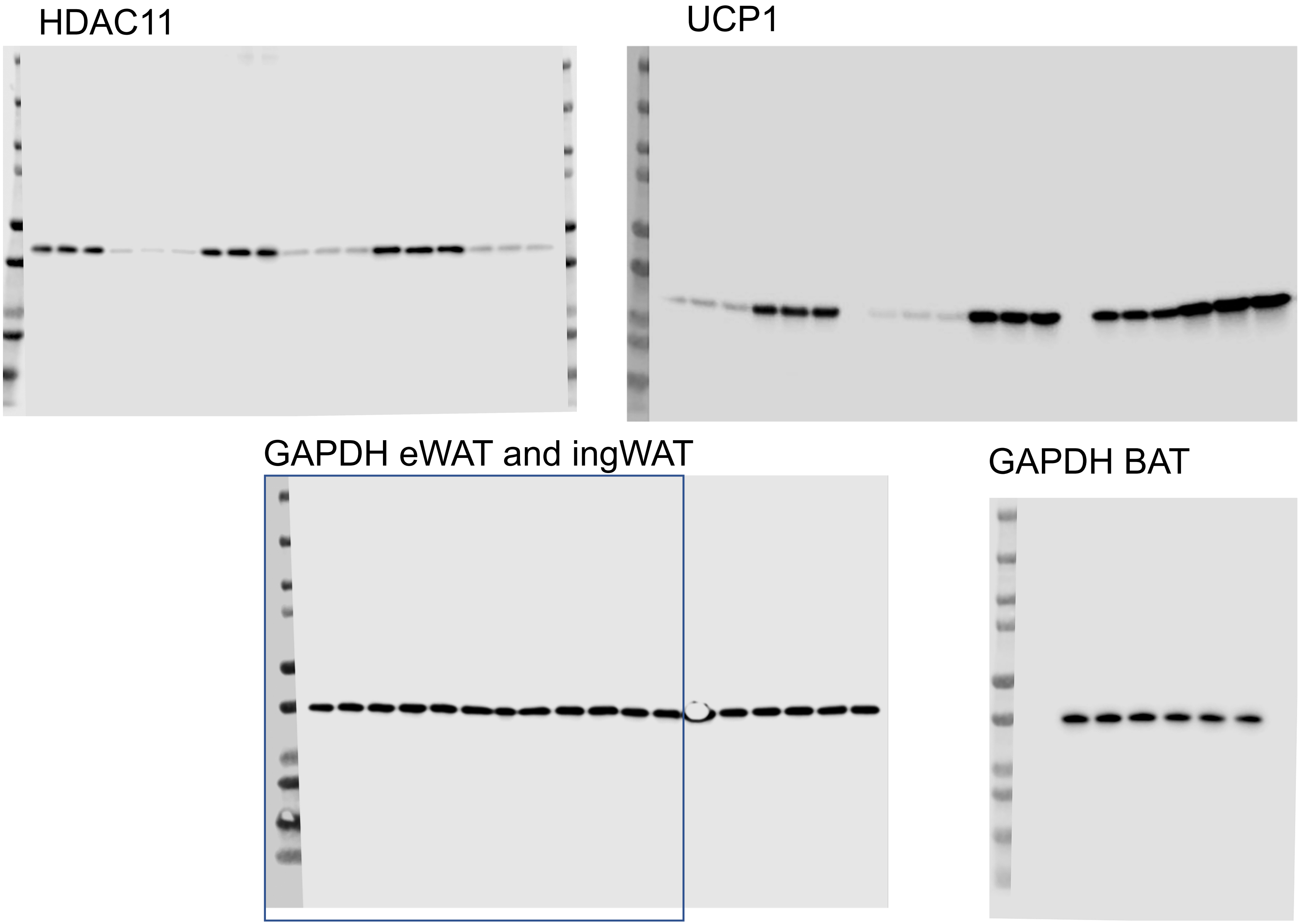

Fig. 1I

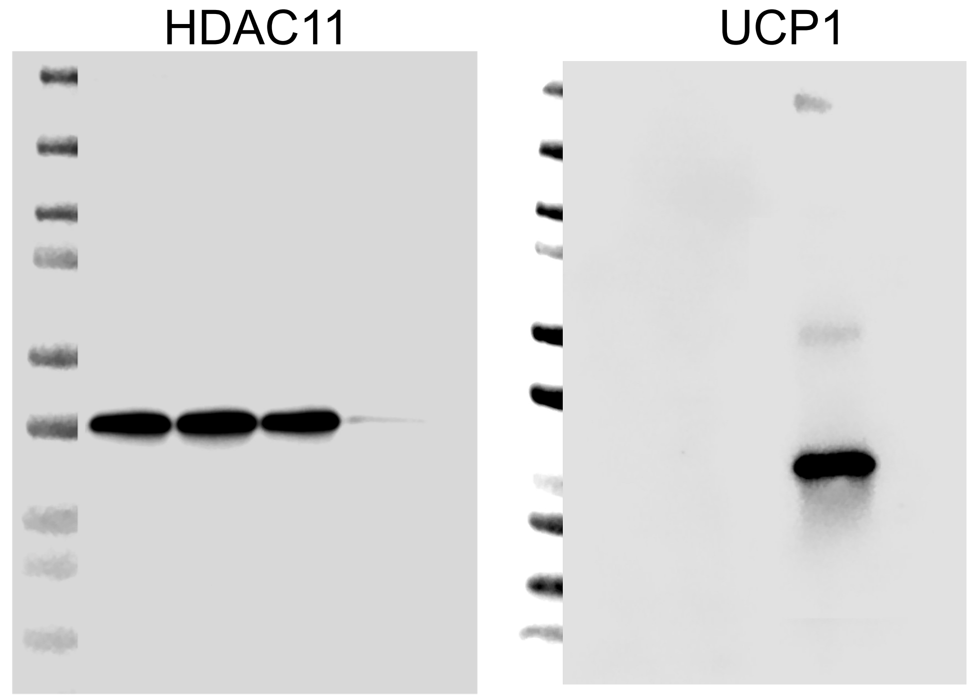

Fig. 2D

UCP1

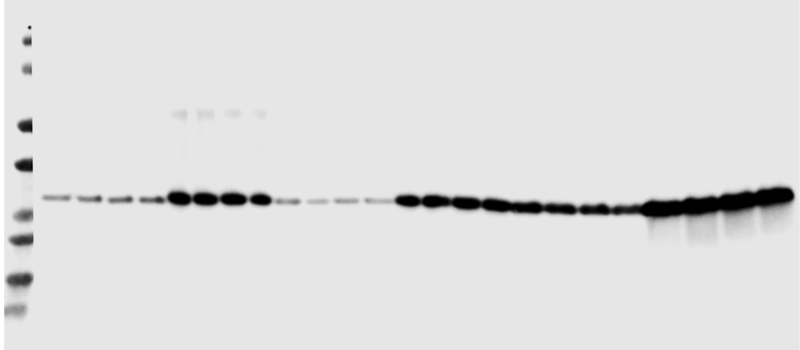

HDAC11

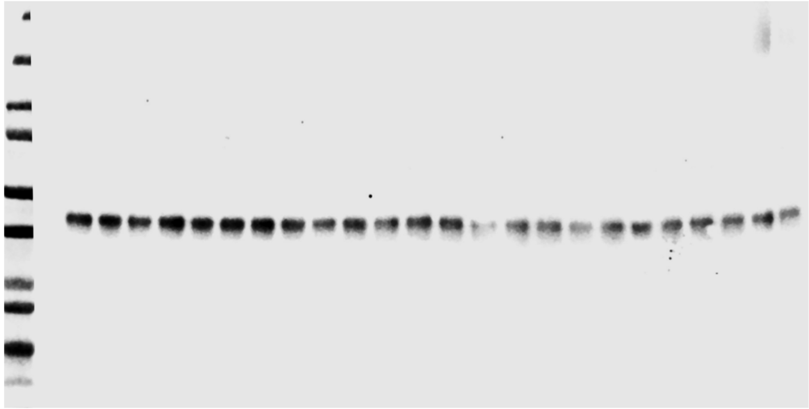

GAPDH

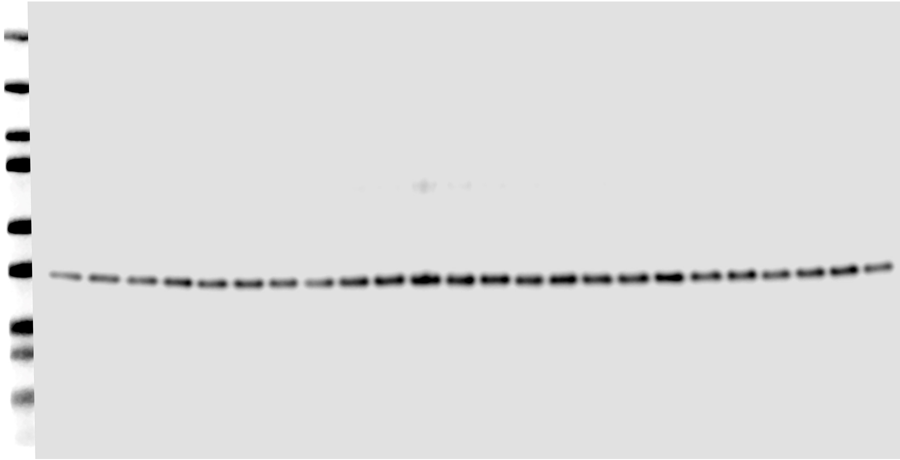

Fig. 3A

UCP1

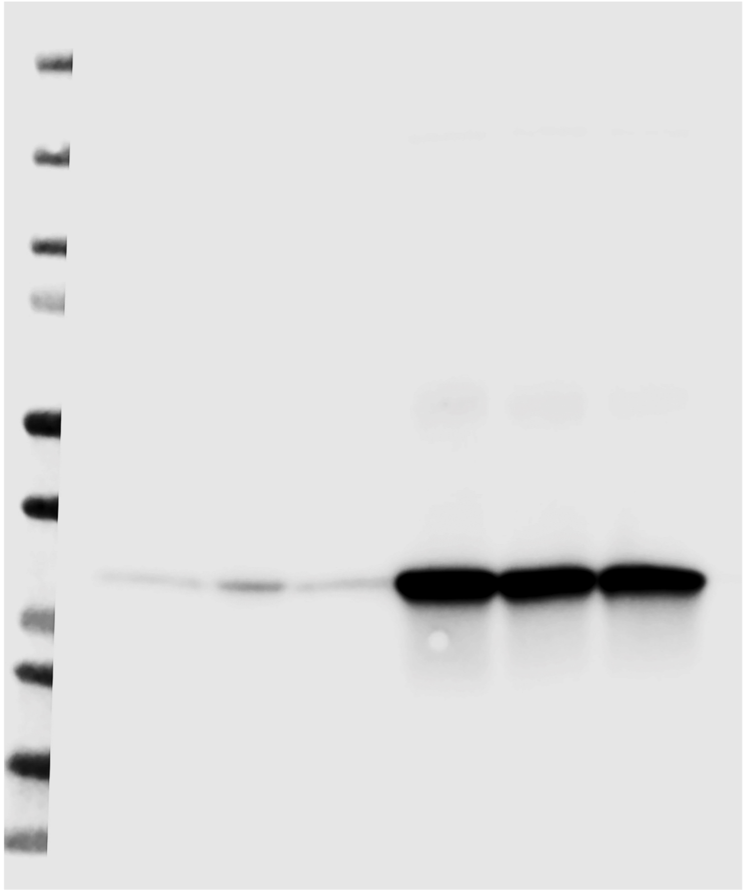

α-Tubulin

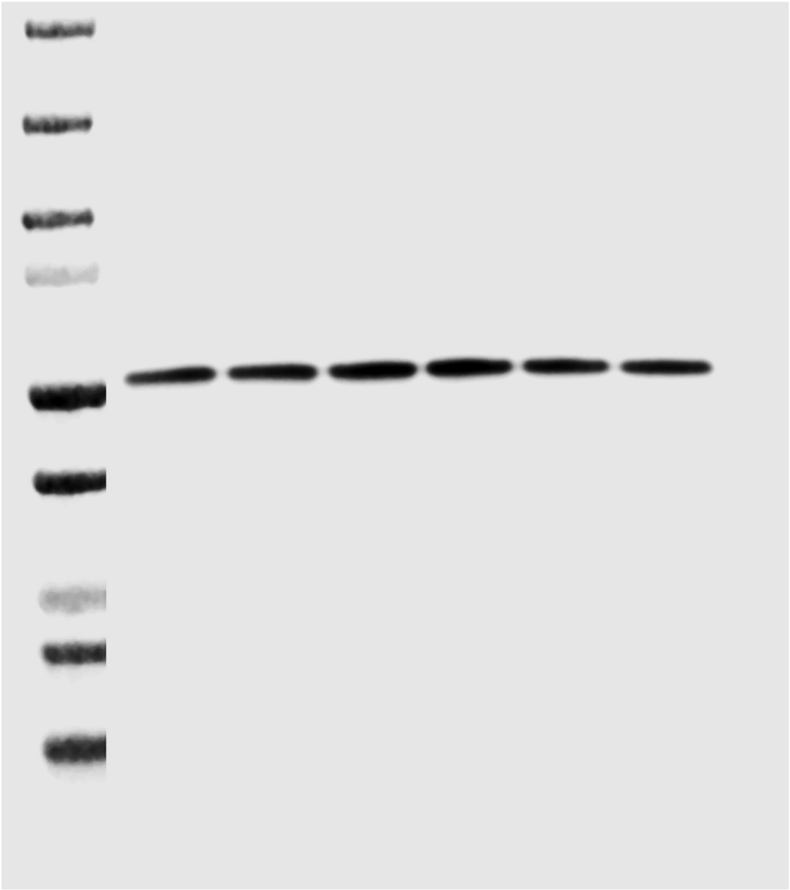

Fig. 3D

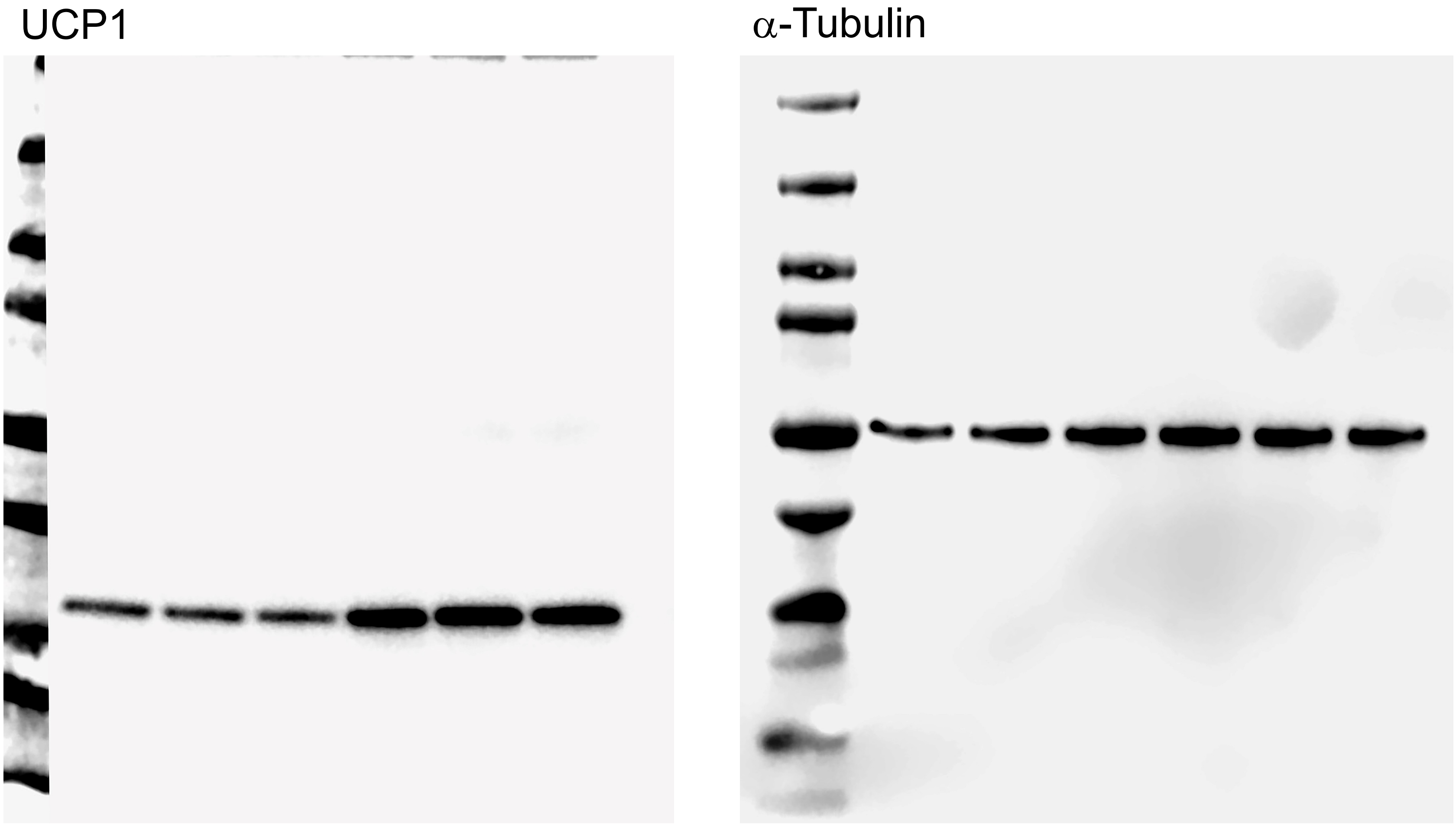

Fig. 3F

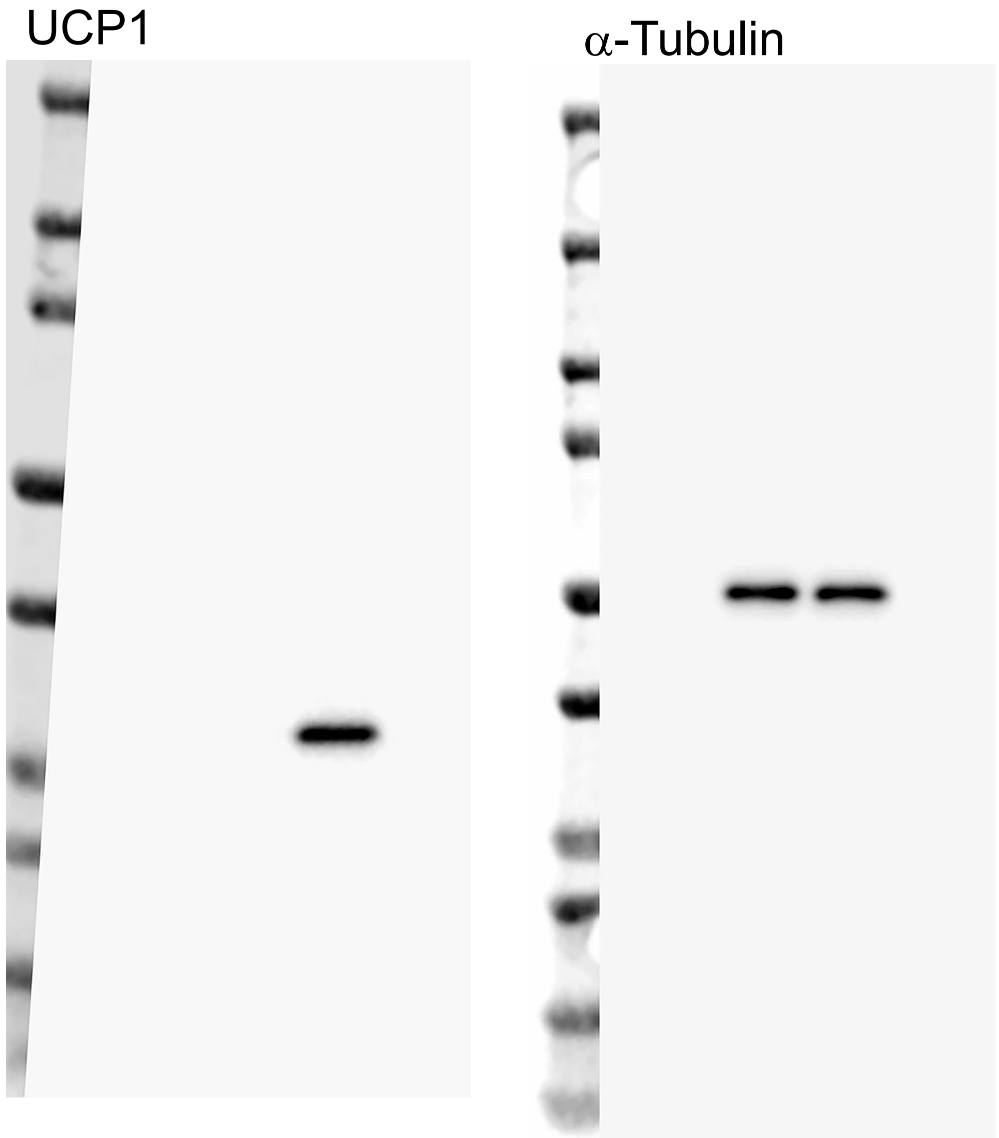

Fig. 3G

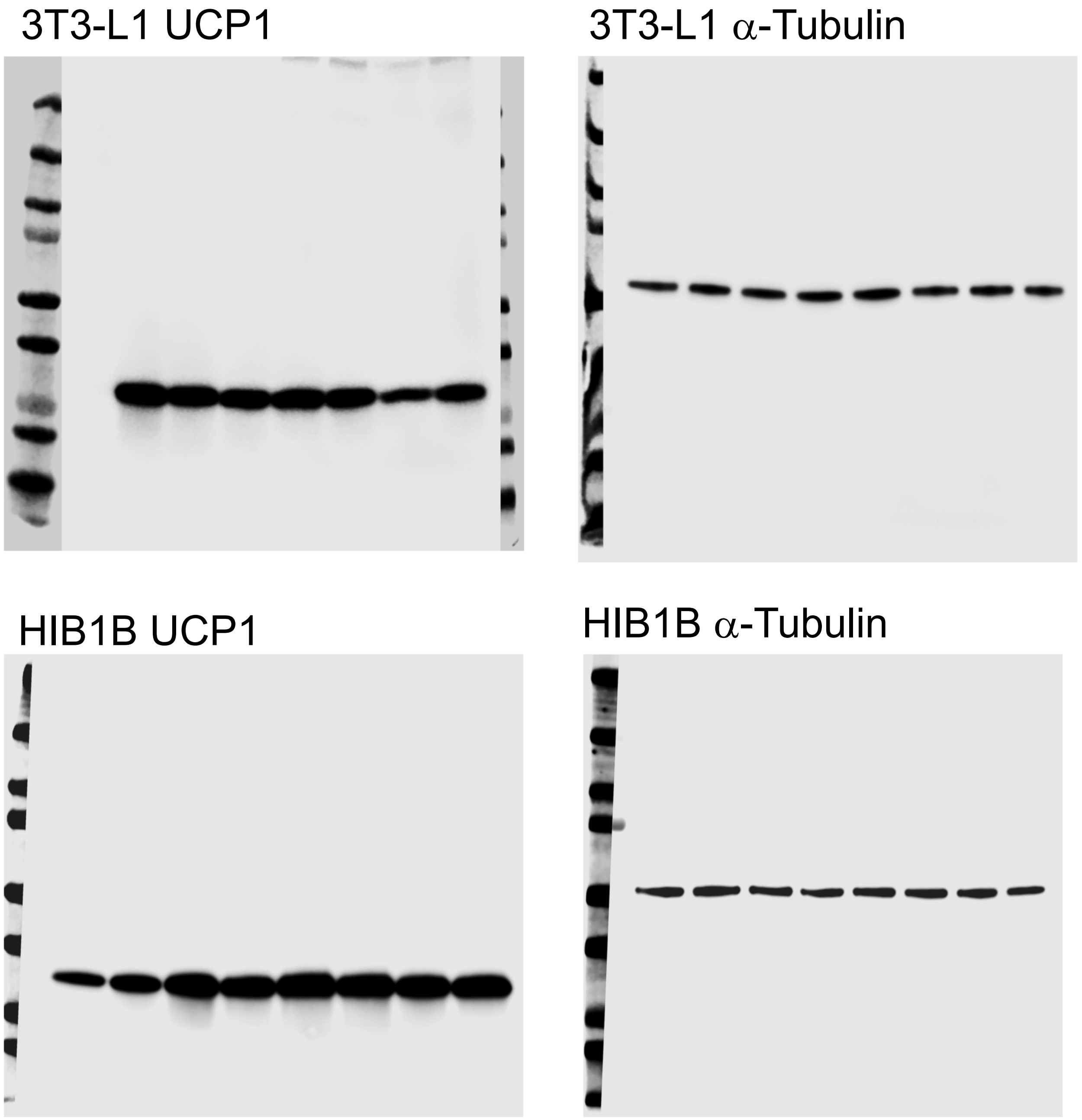

Fig. 3H

UCP1

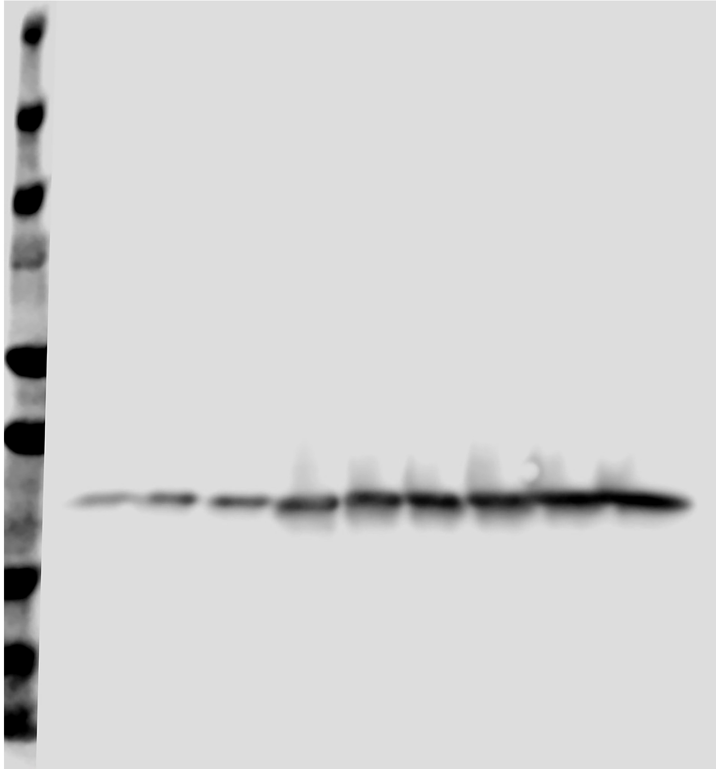

α-Tubulin

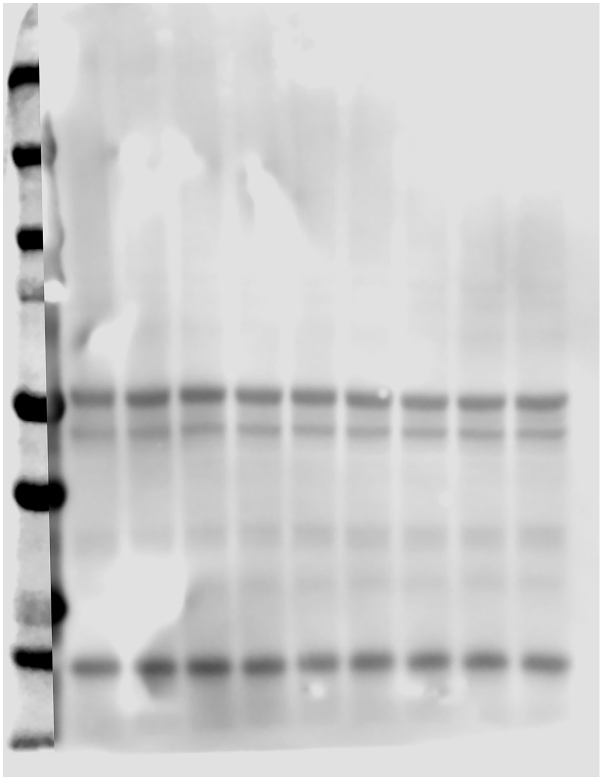

Fig. 4A

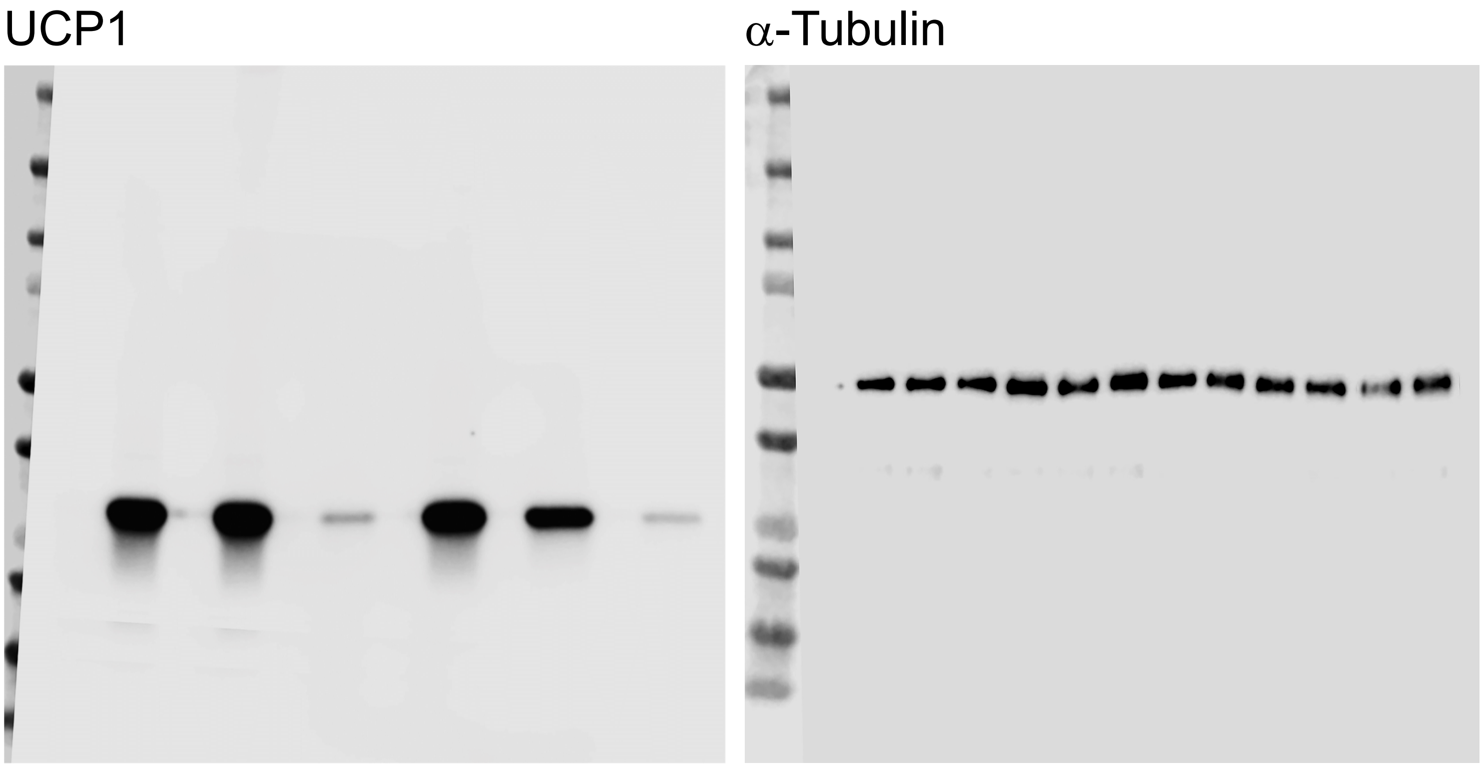

Fig. 5C

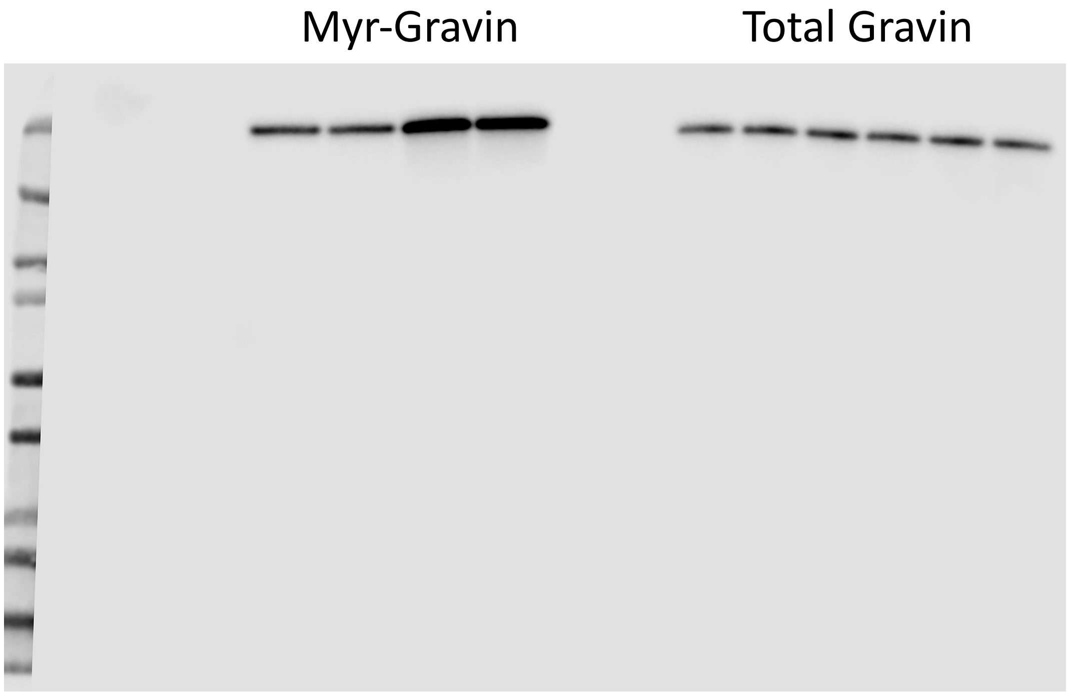

Fig. 6B

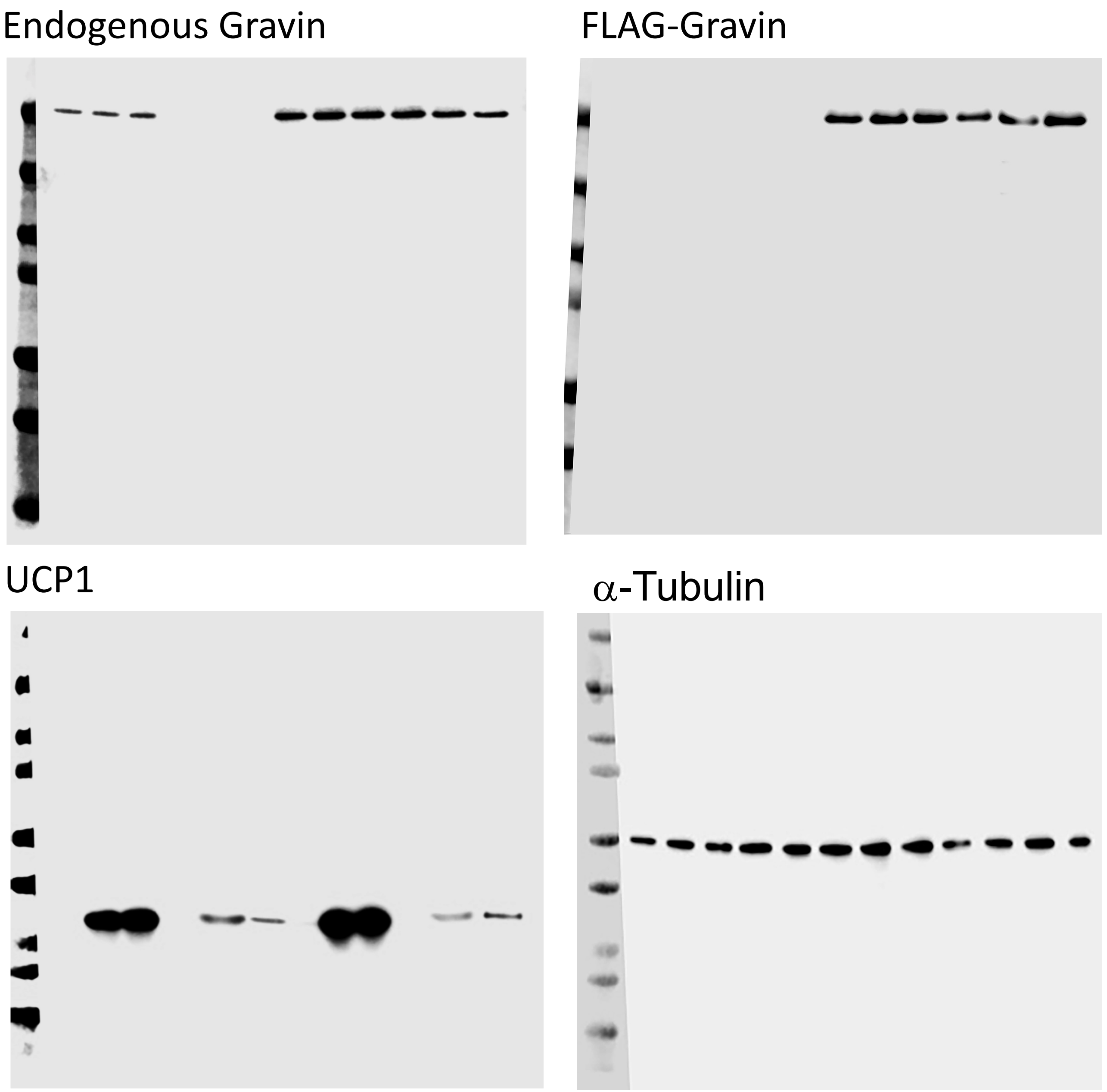

Fig. 6C

P-PKA Substrates

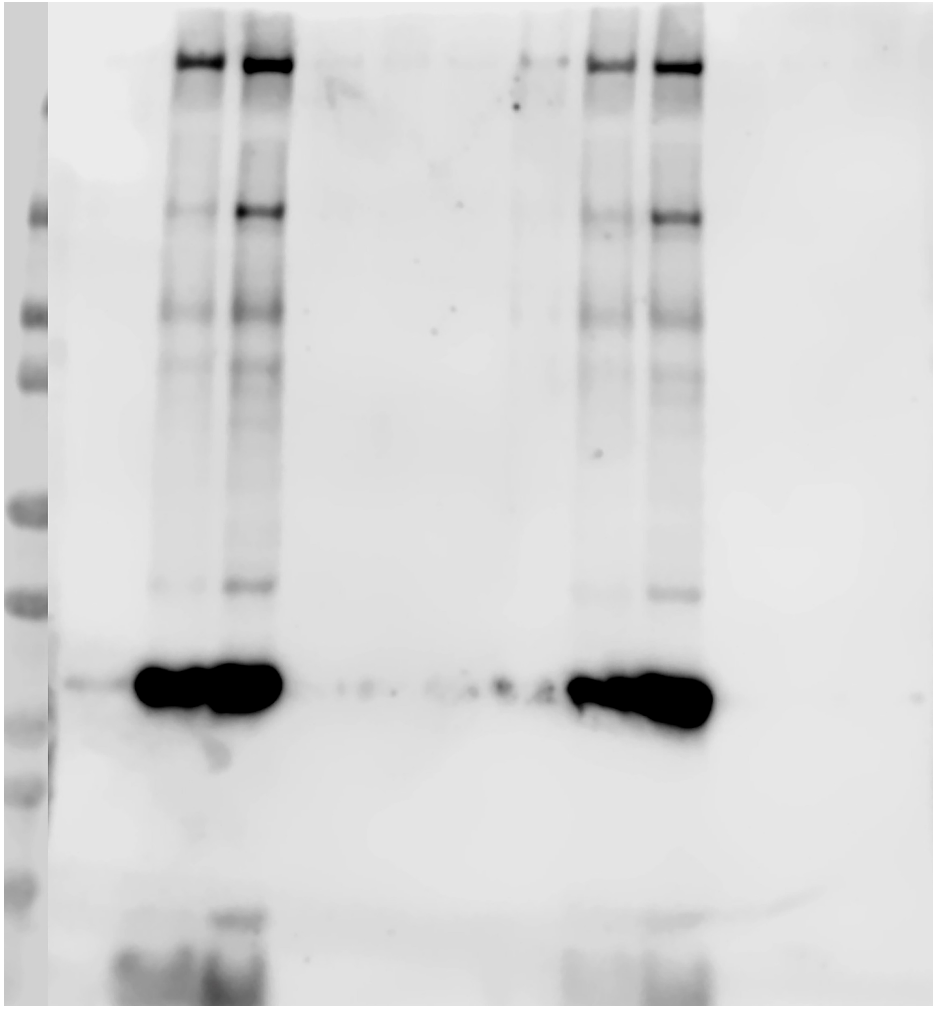

Fig. 7B

UCP1

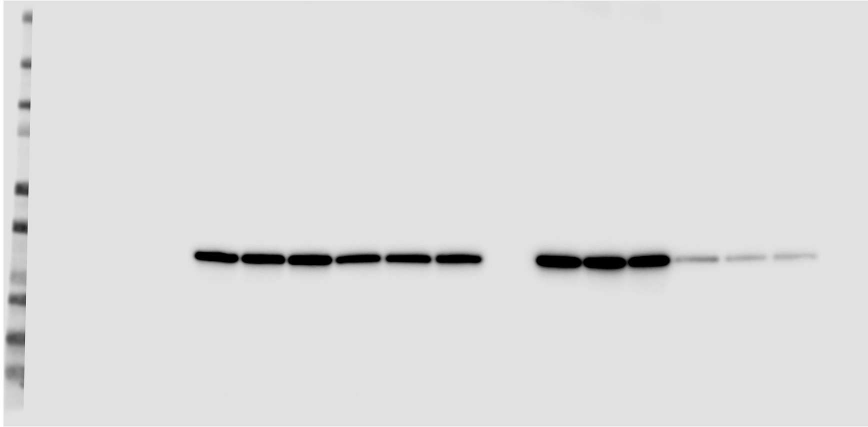

α-Tubulin

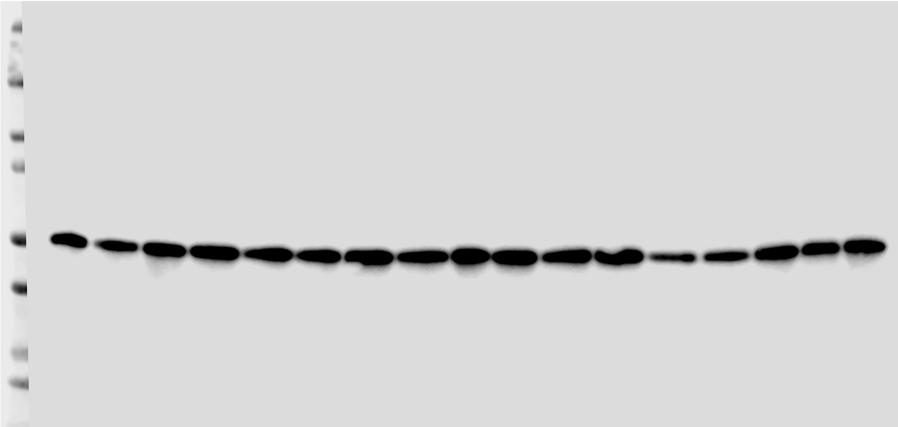

Fig. 7D

P-PKA Substrates

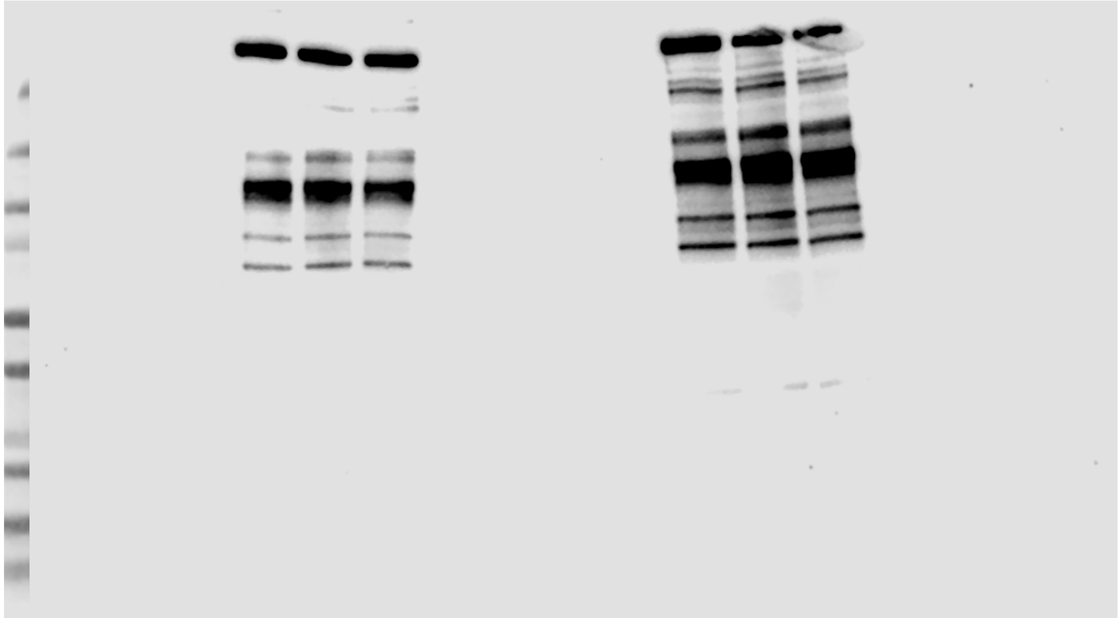

Fig. 8B

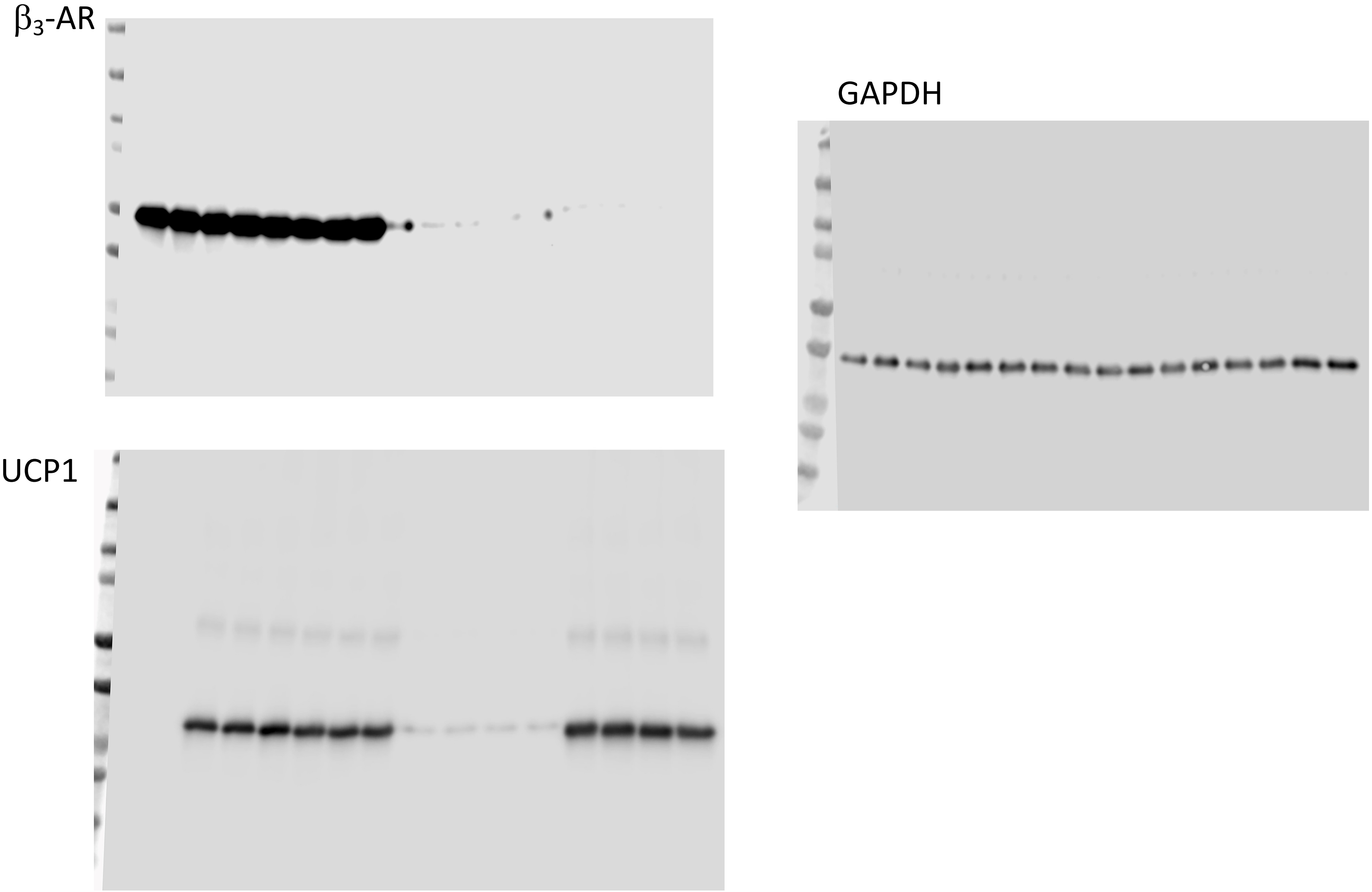

Fig. 8D

P-PKA Substrates

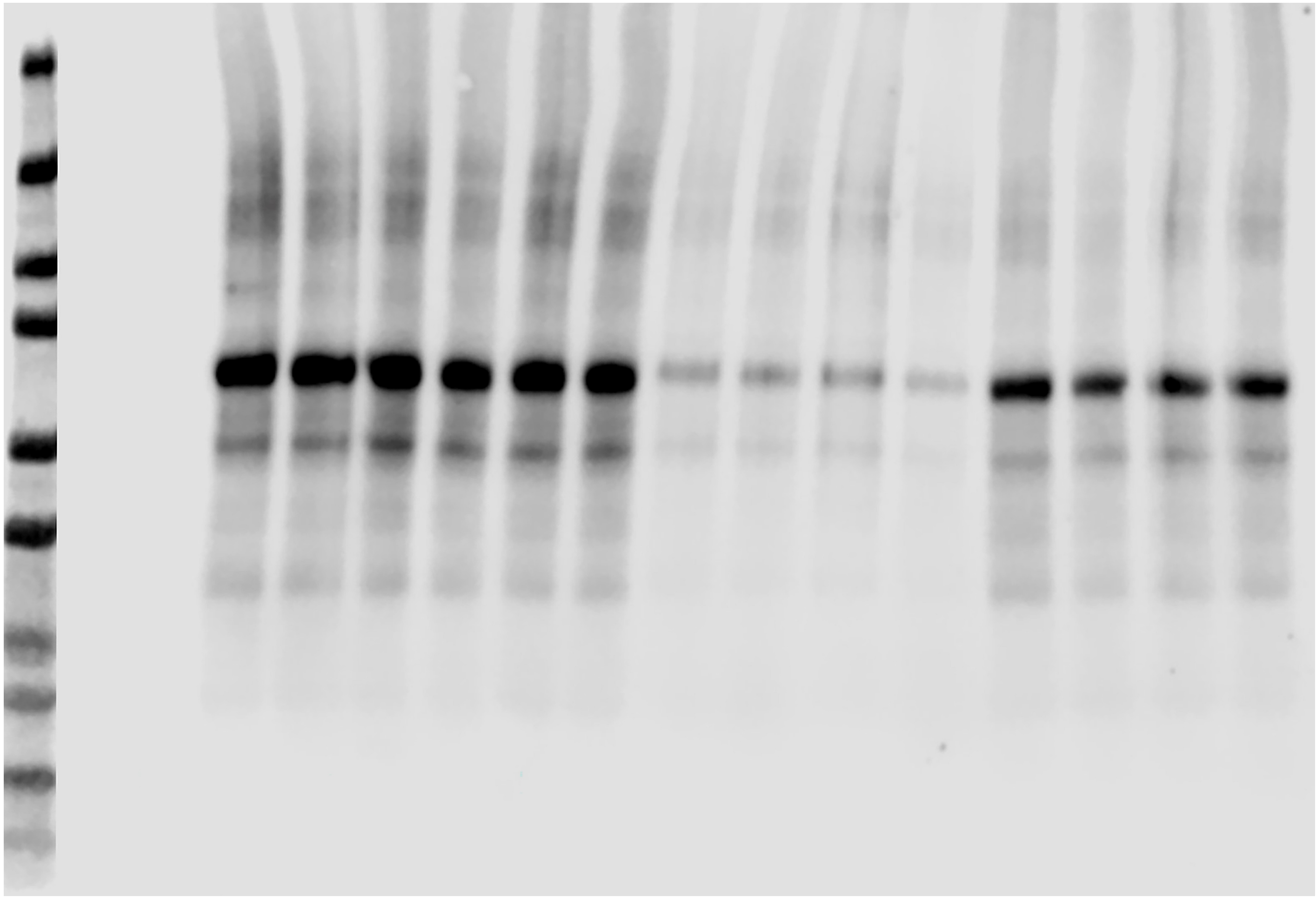

Fig. 8F

β_3-AR

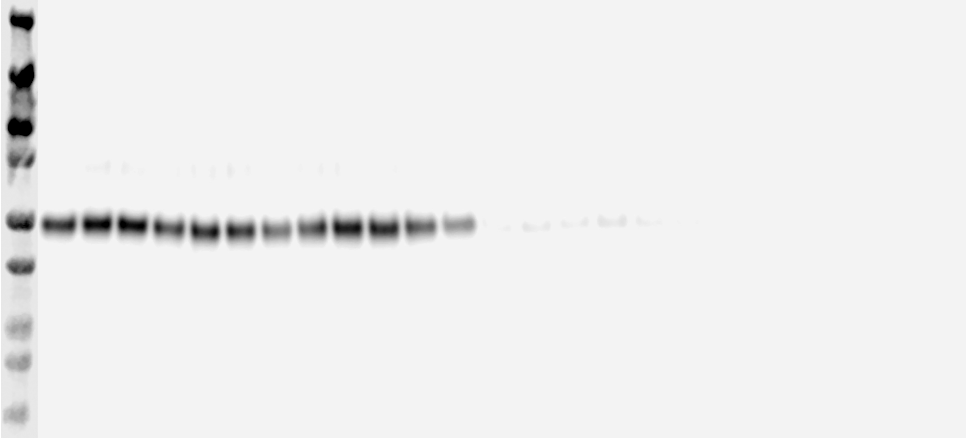

GAPDH

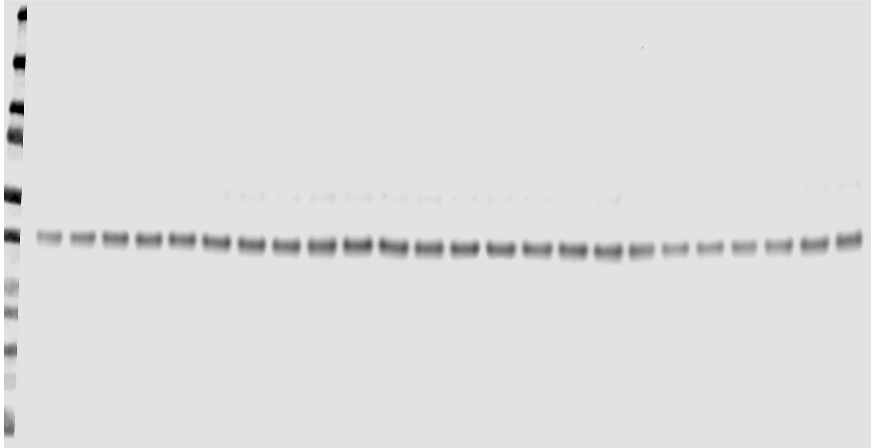

UCP1

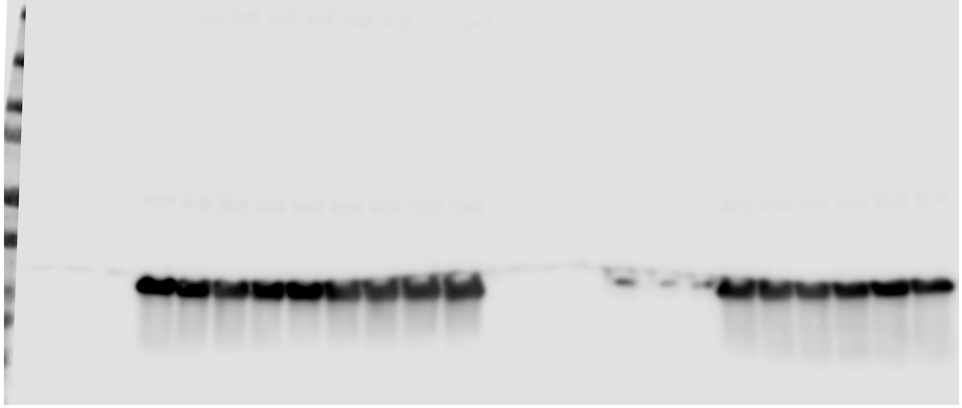

Fig. 8H

P-PKA Substrates

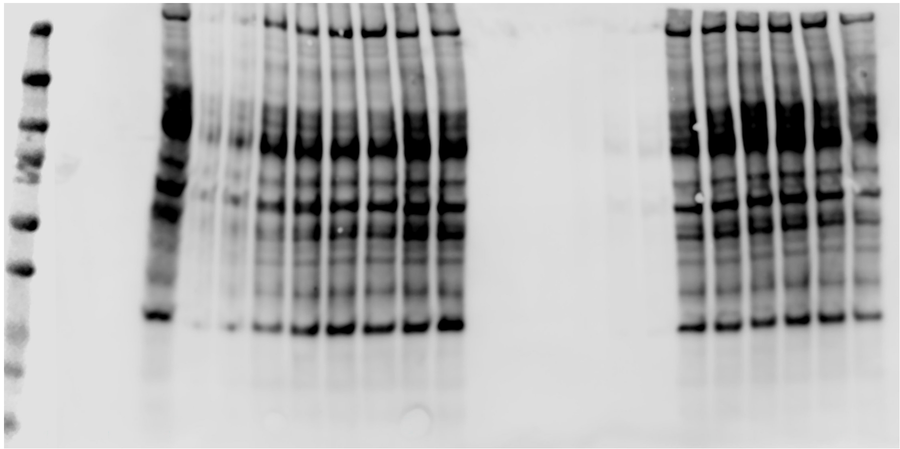

Fig. 9B

β_3-AR

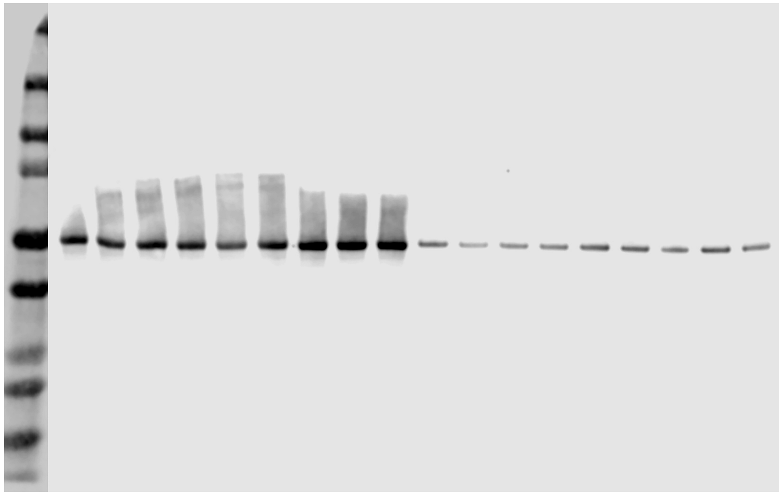

UCP1

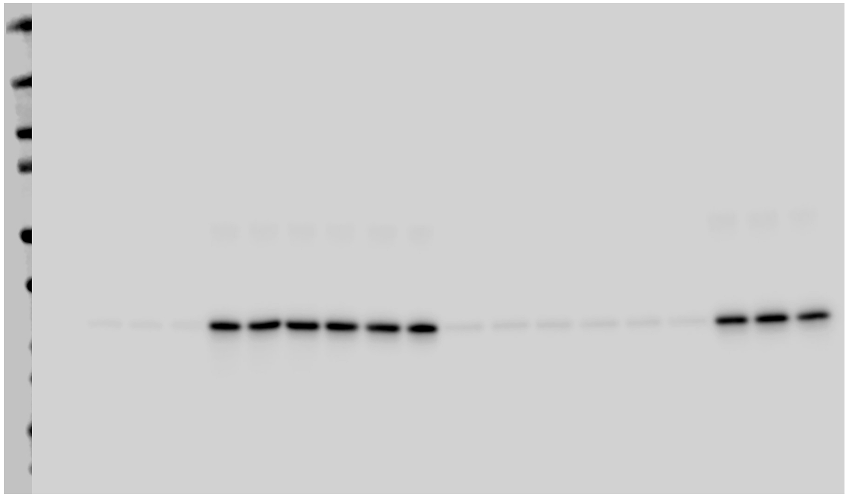

GAPDH

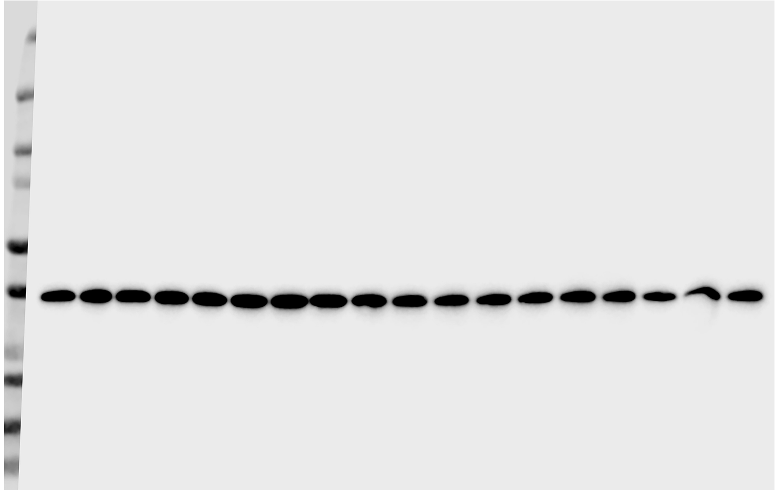

Fig. 9C

β_3-AR

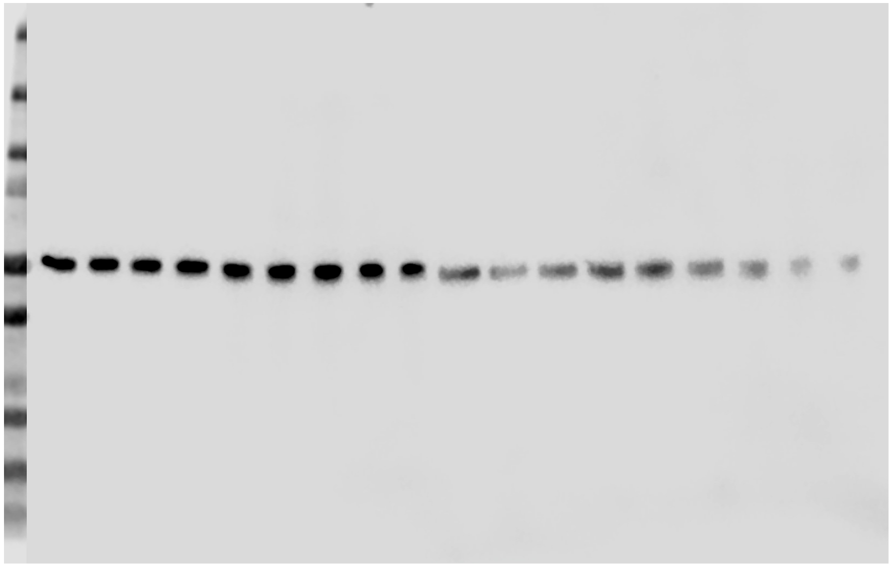

GAPDH

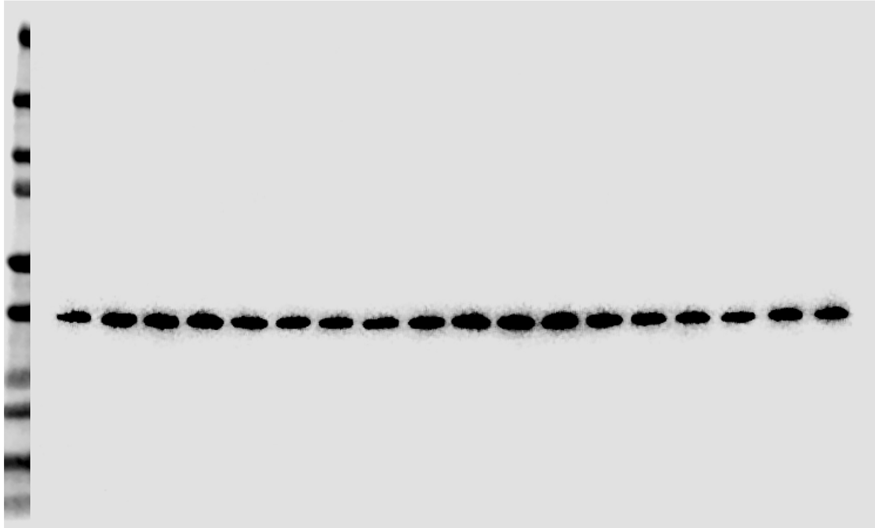

UCP1

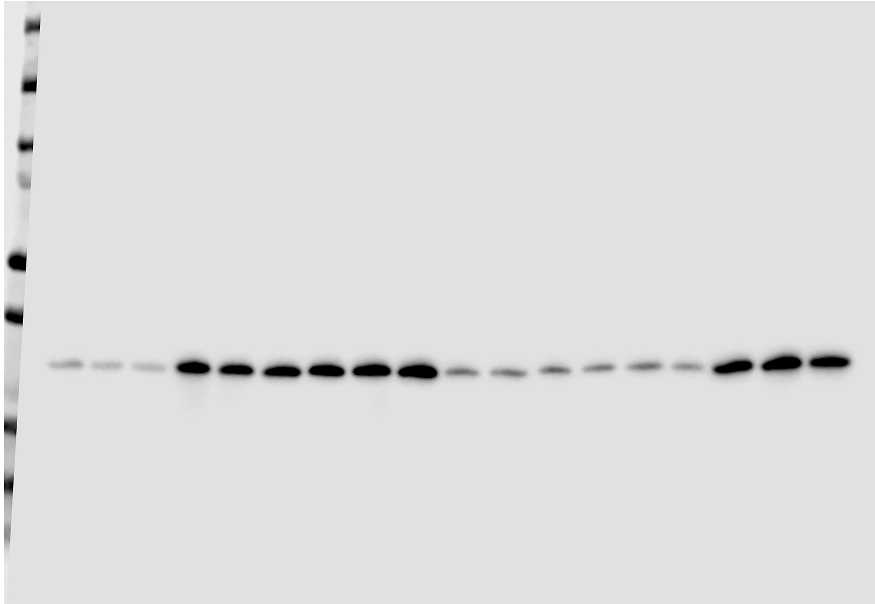

Fig. 9D

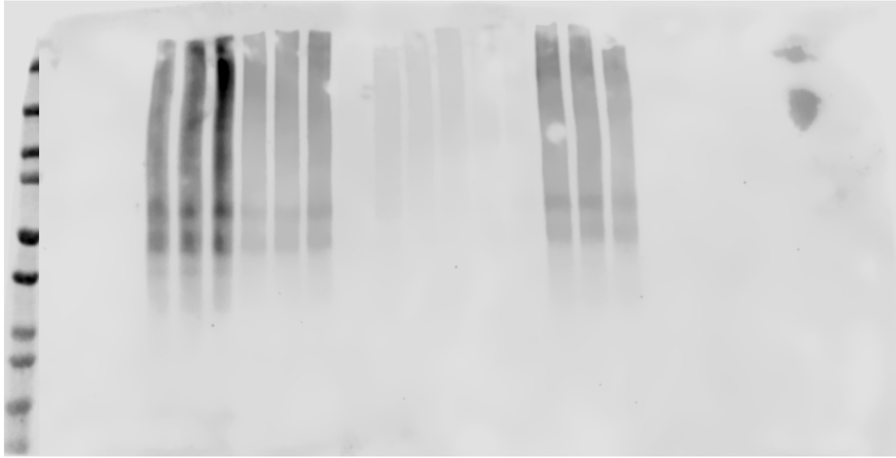

Fig. 9E

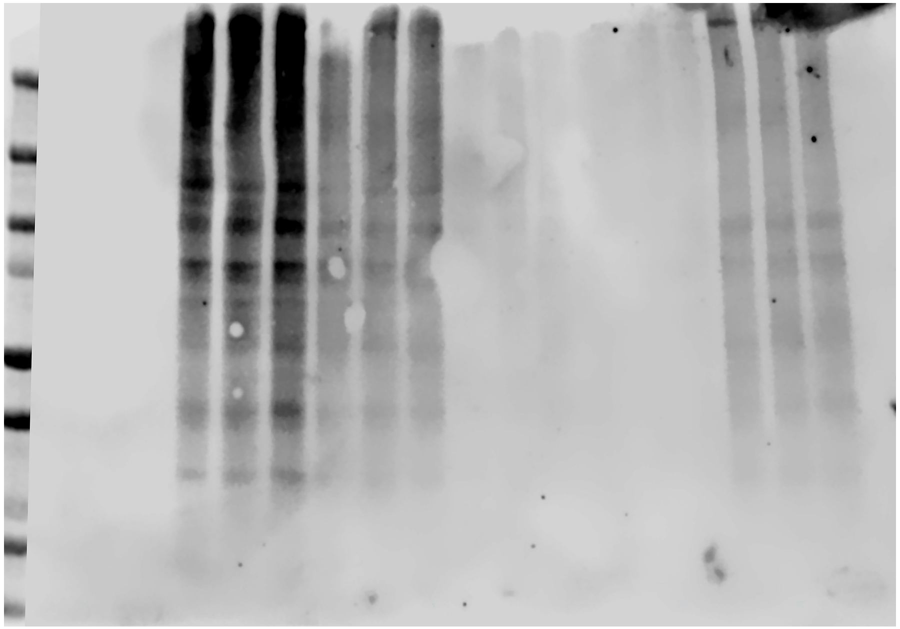

Fig. 10B

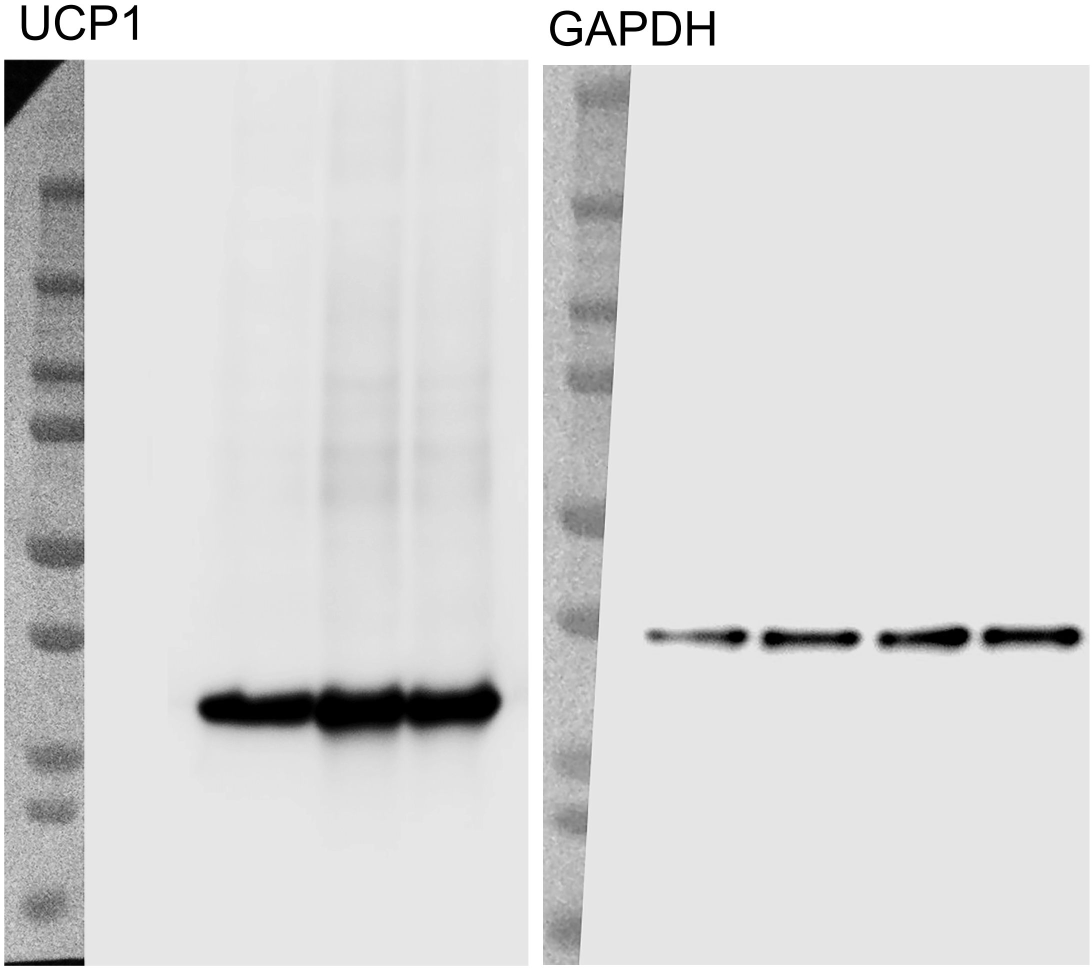

Fig. 10C

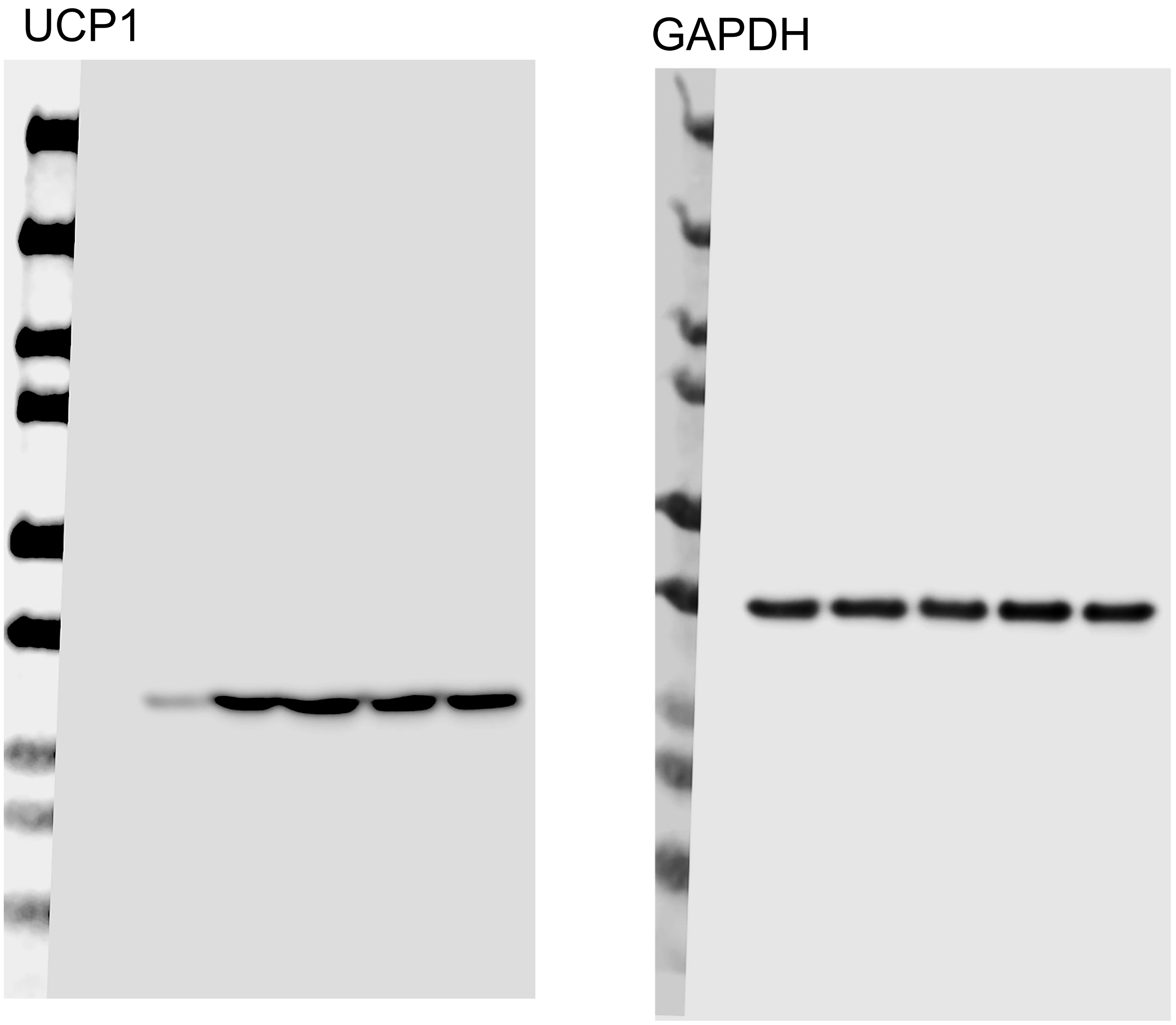

Fig. 10E

β_3-AR

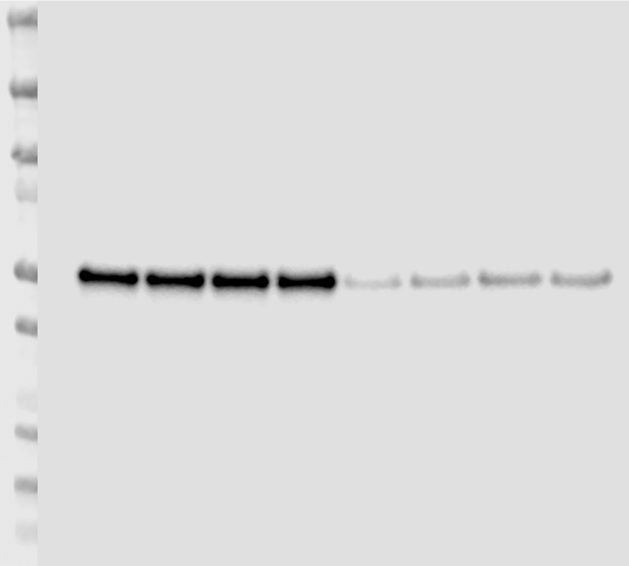

GAPDH

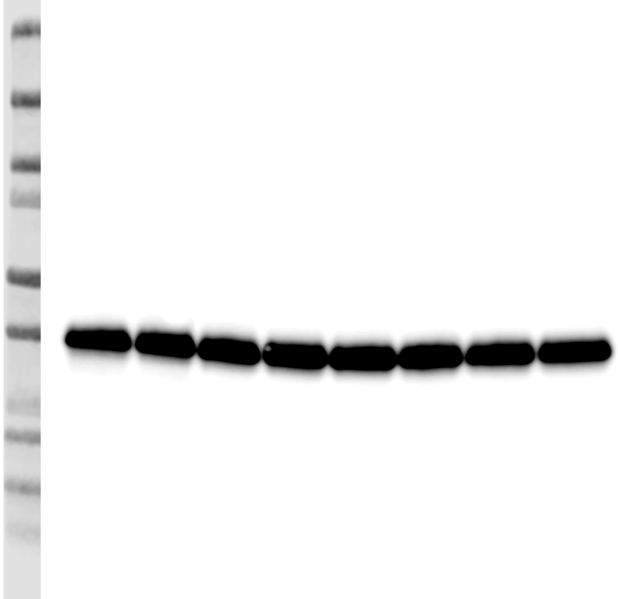

UCP1

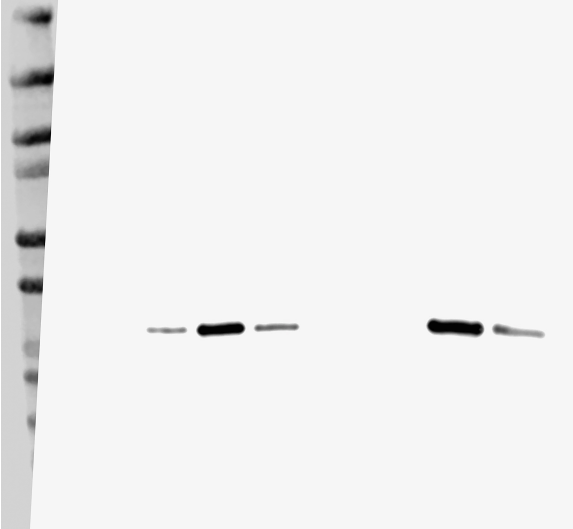

Fig. 10G

P-PKA Substrates

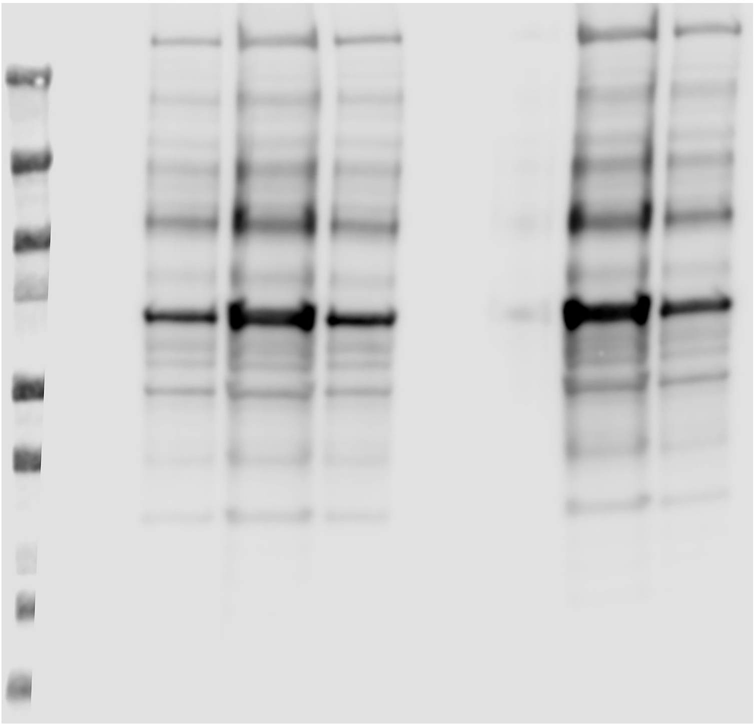

Supplemental Figure 2

UCP1

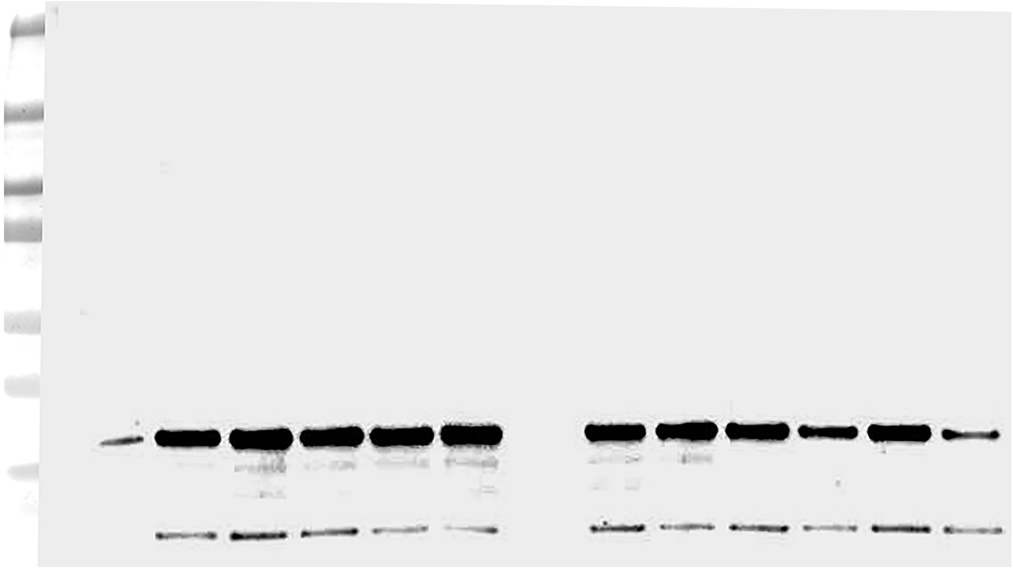

GAPDH

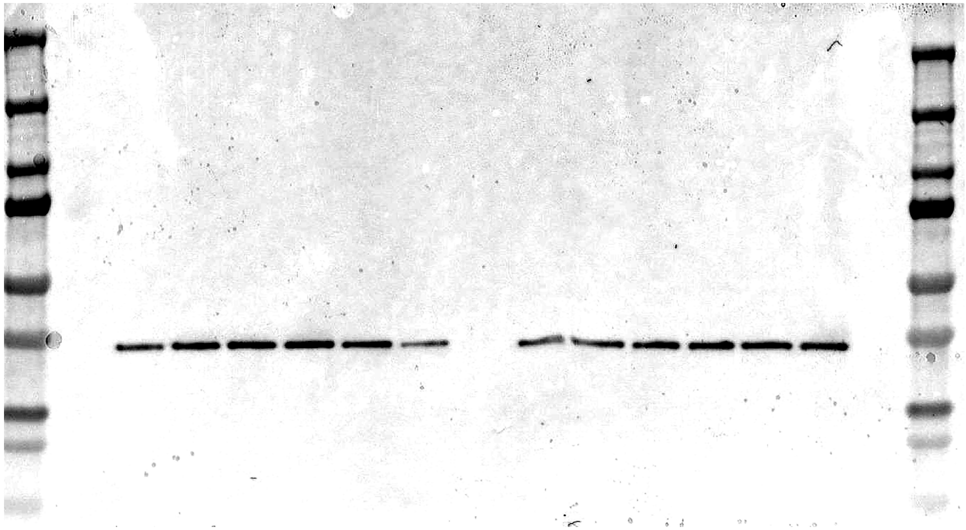

Supplemental Figure 3B

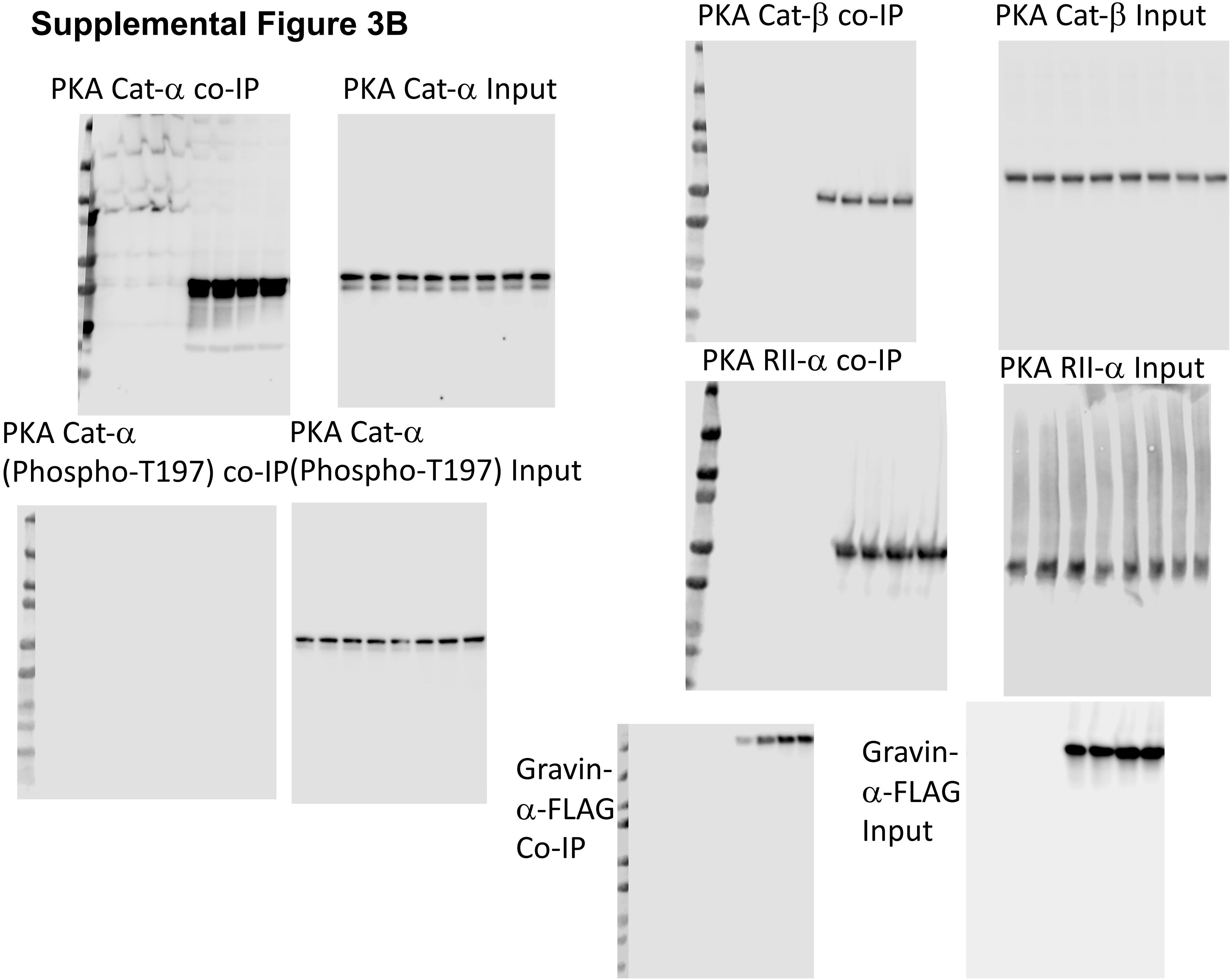

Supplemental Figure 4A

UCP1

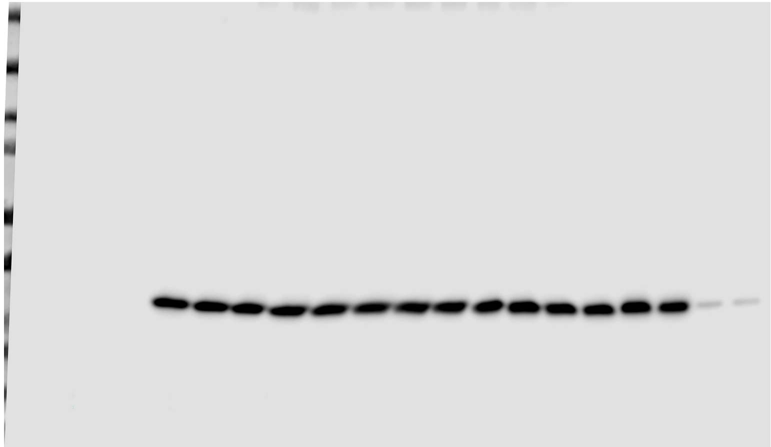

GAPDH

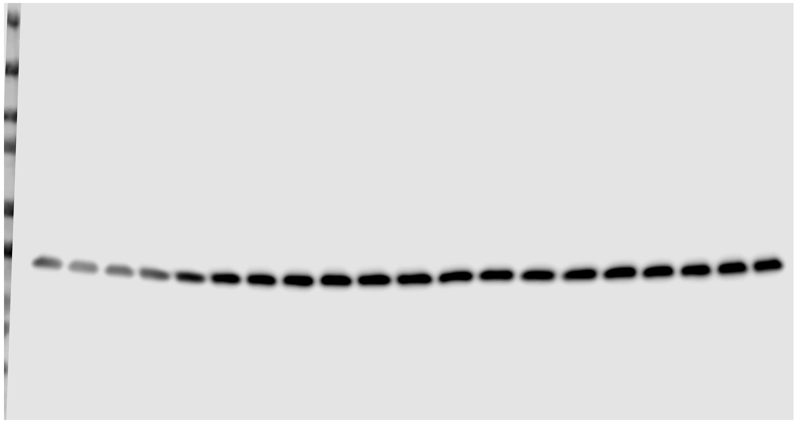

Supplemental Figure 4C

P-PKA Substrates

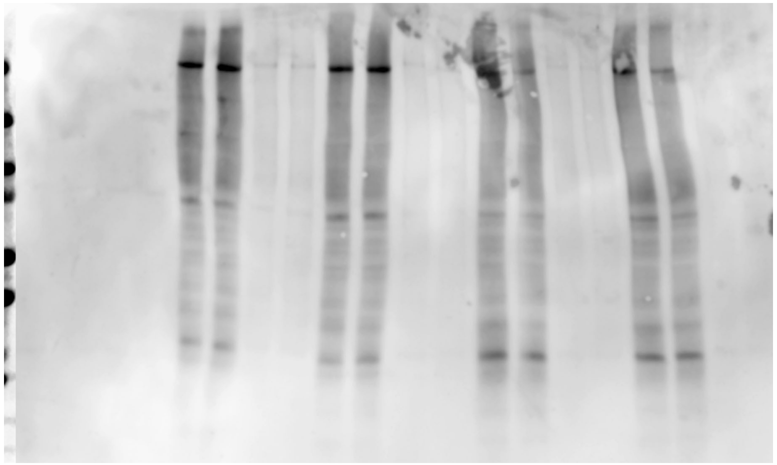

Supplemental Figure 5B

UCP1

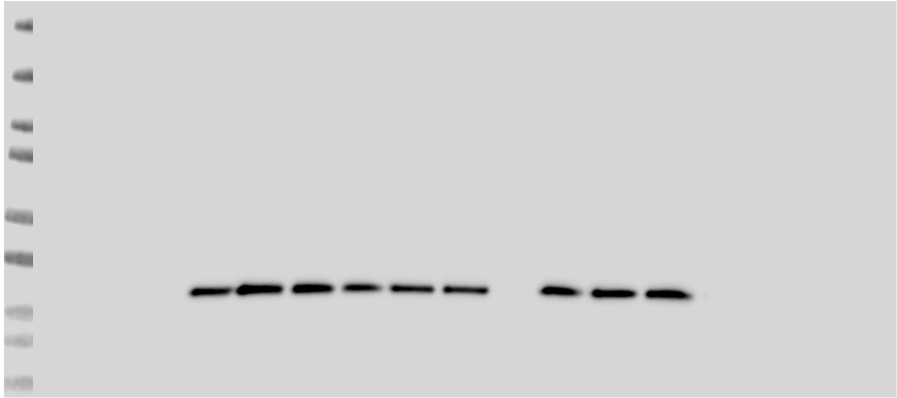

P-PKA Substrates

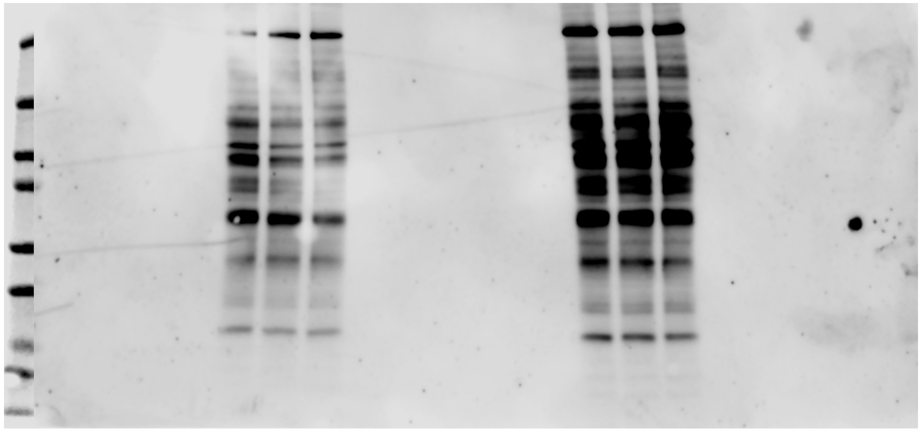

GAPDH

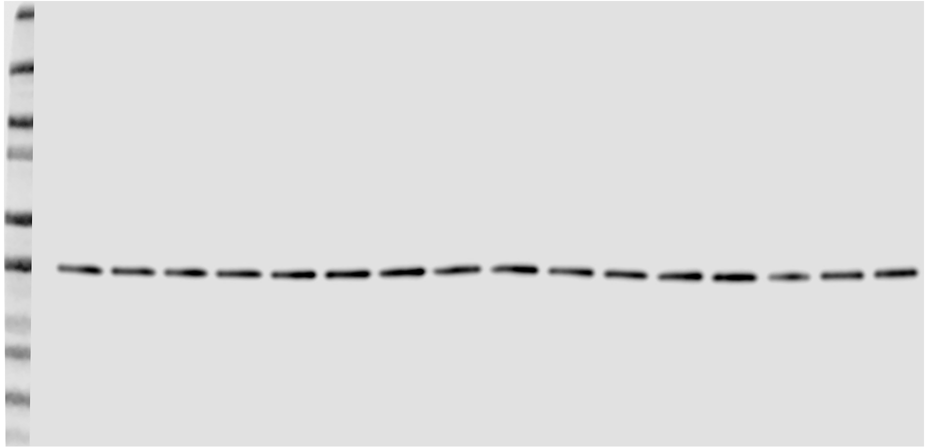

Supplemental Figure 6B

β_2-AR

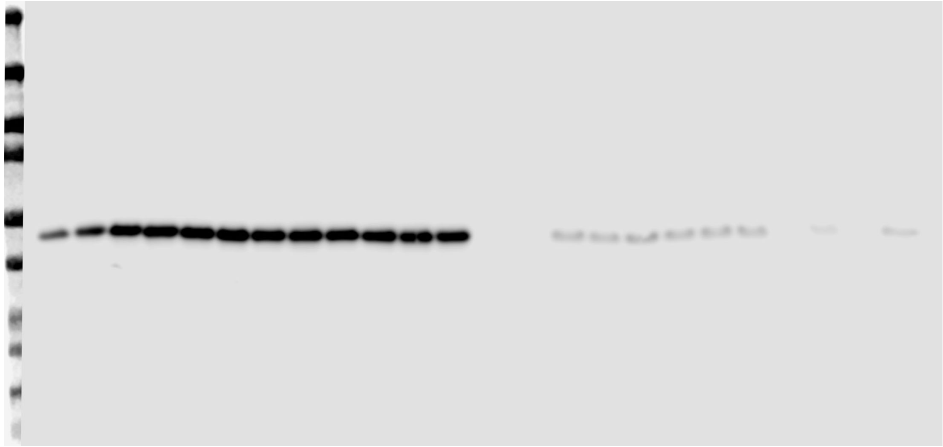

GAPDH

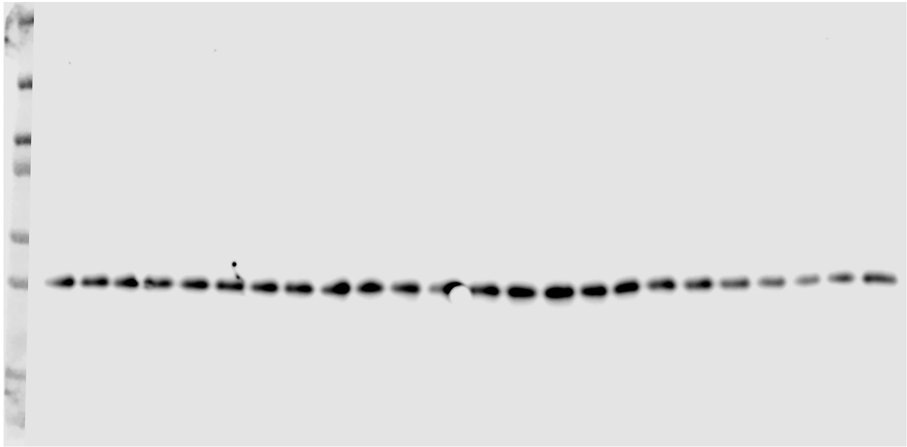

UCP1

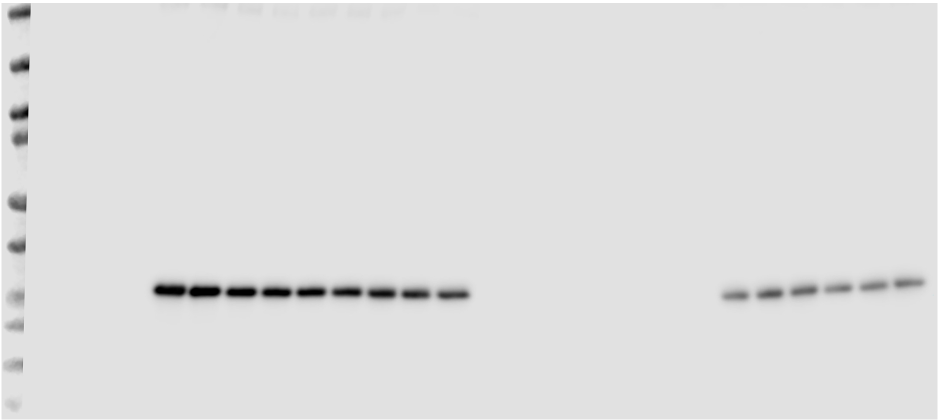

Supplemental Figure 6D

P-PKA Substrates

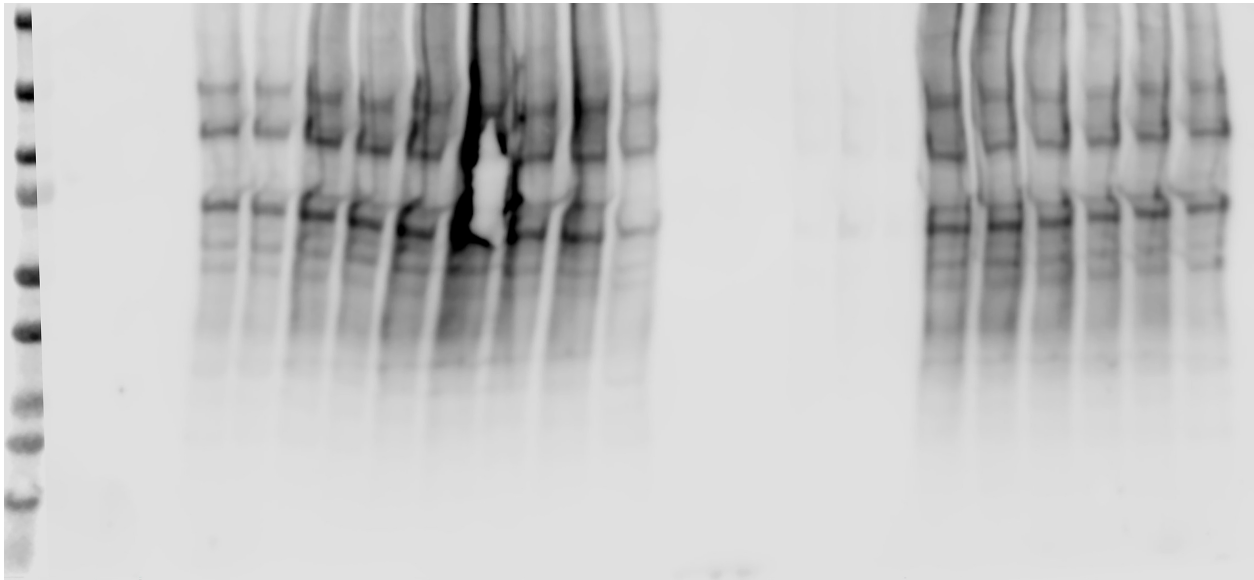

Supplemental Figure 6F

GαS

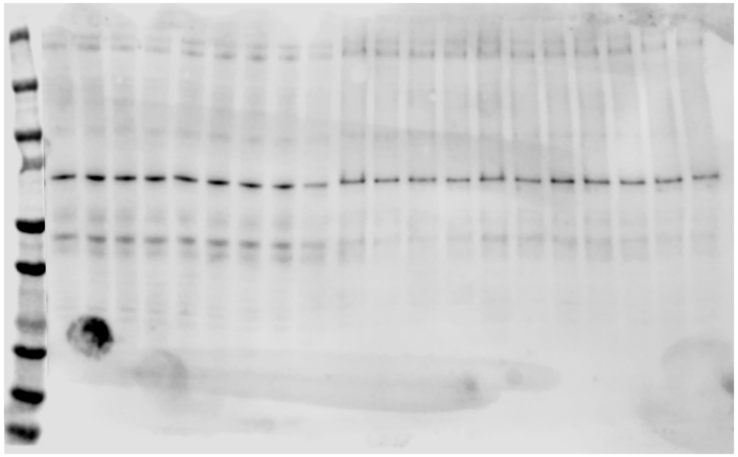

UCP1

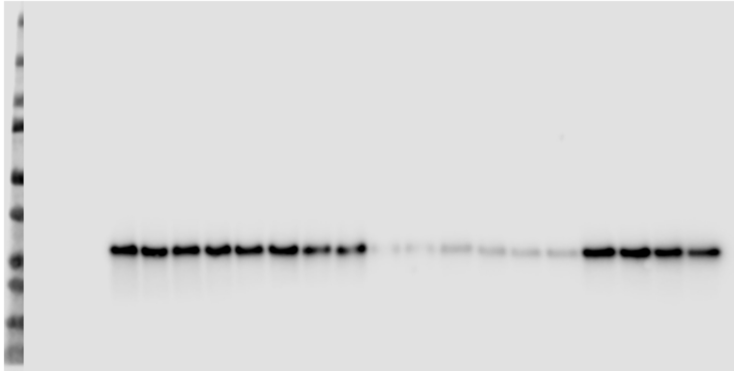

β_3-AR

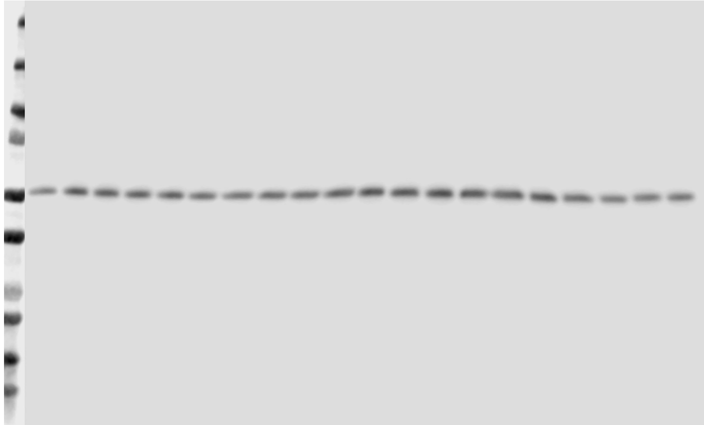

β_2-AR

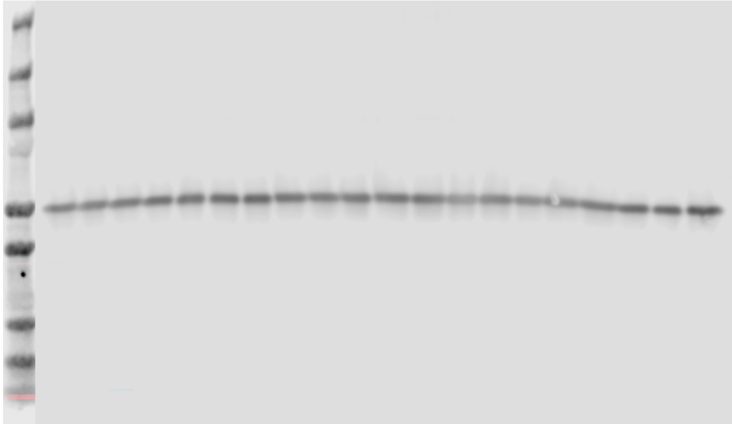

GAPDH

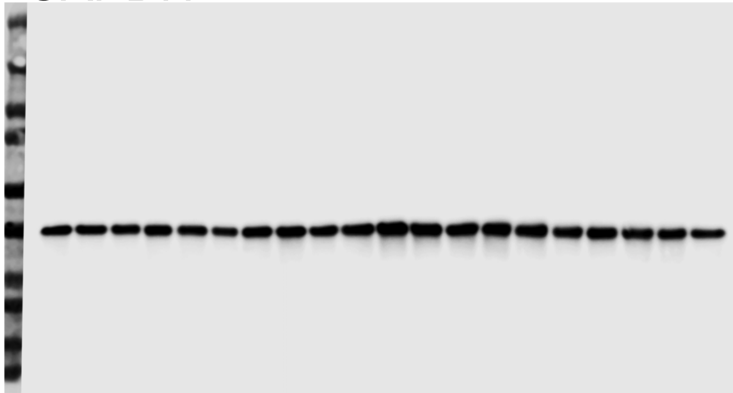

Supplemental Figure 6G

P-PKA Substrates

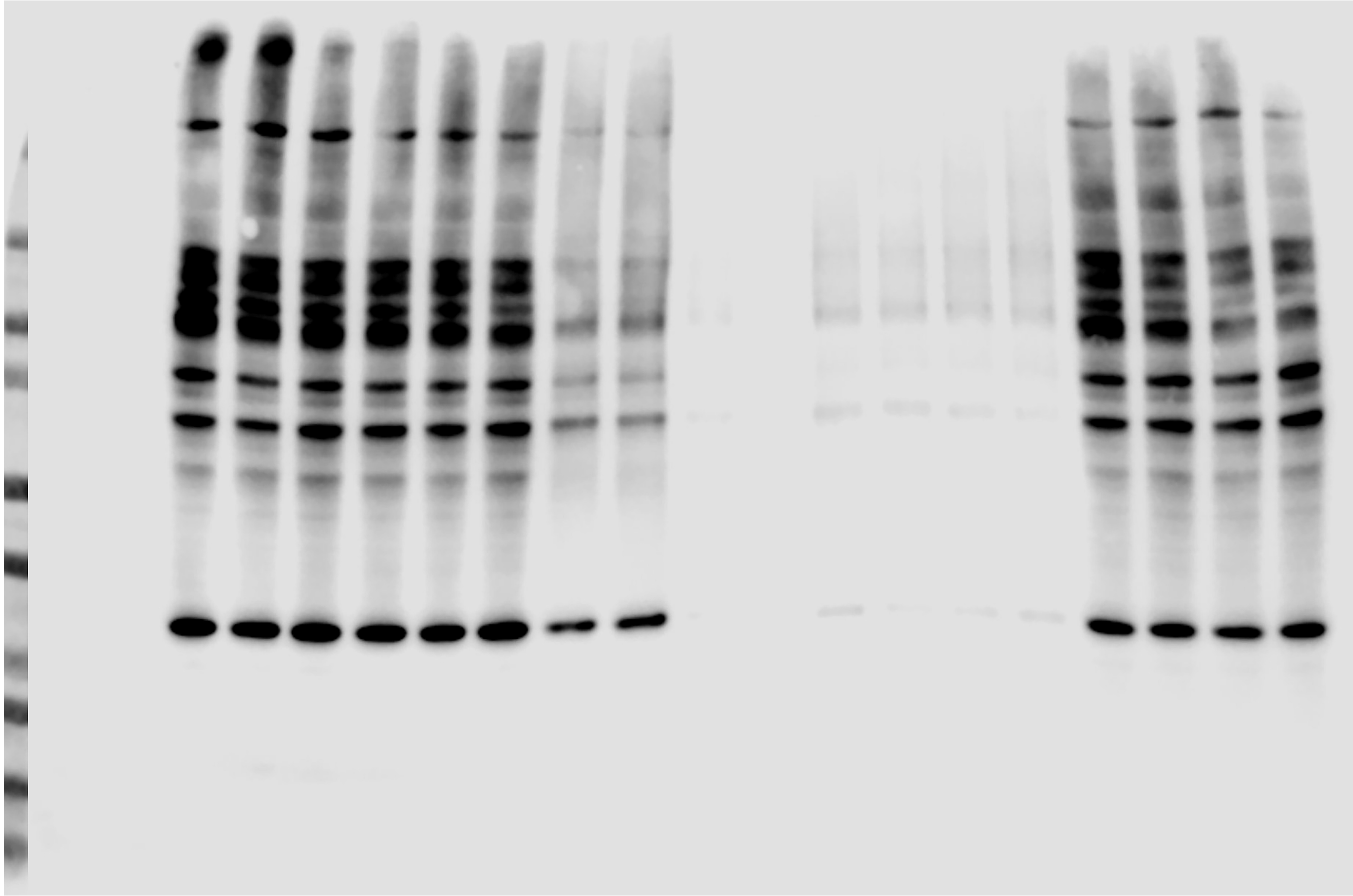

Supplemental Figure 7A

UCP1

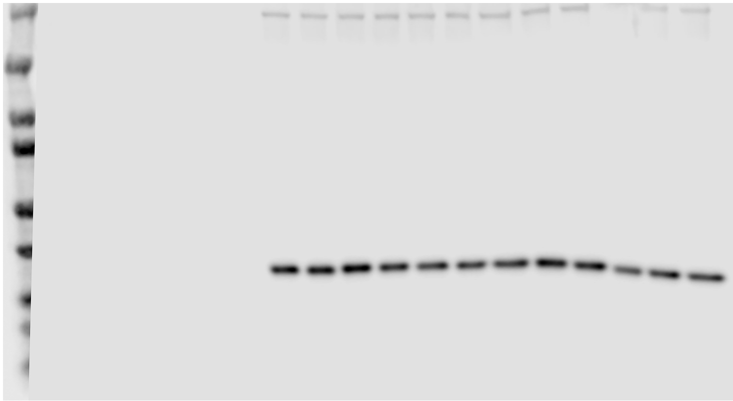

GAPDH

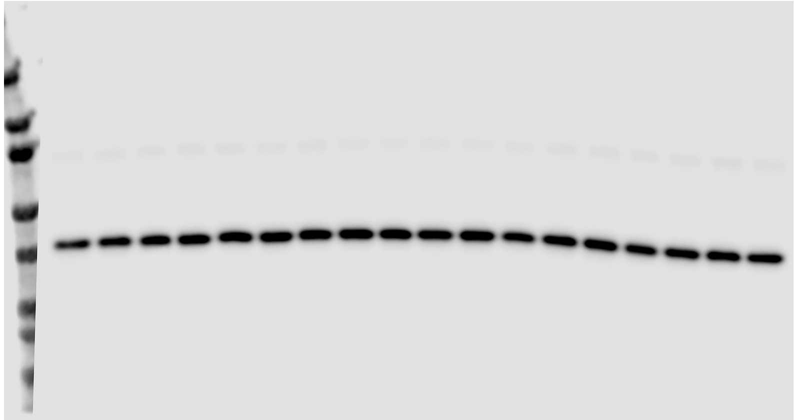

P-PKA Substrates

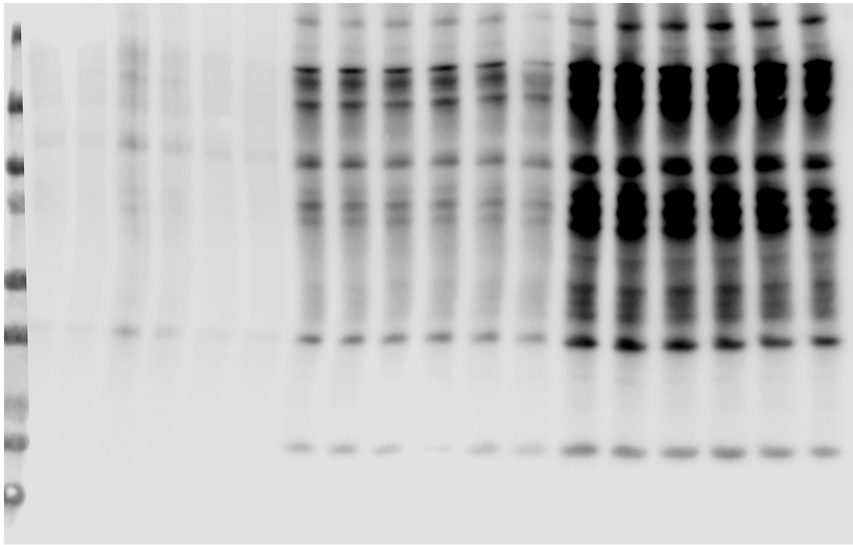

Supplemental Figure 7C

UCP1

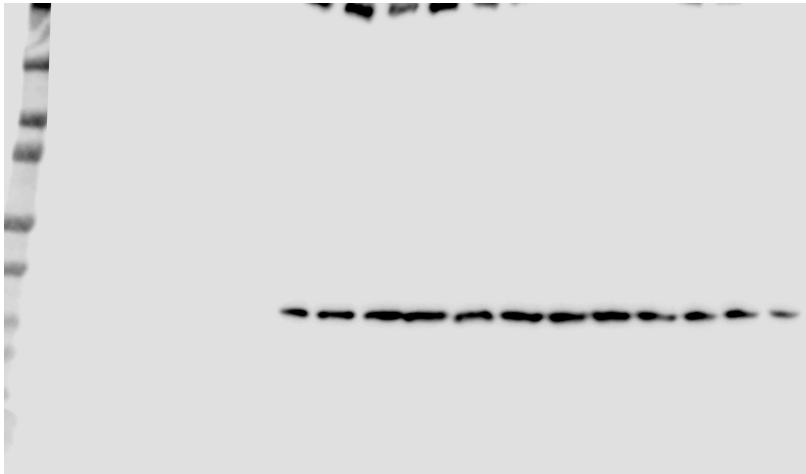

GAPDH

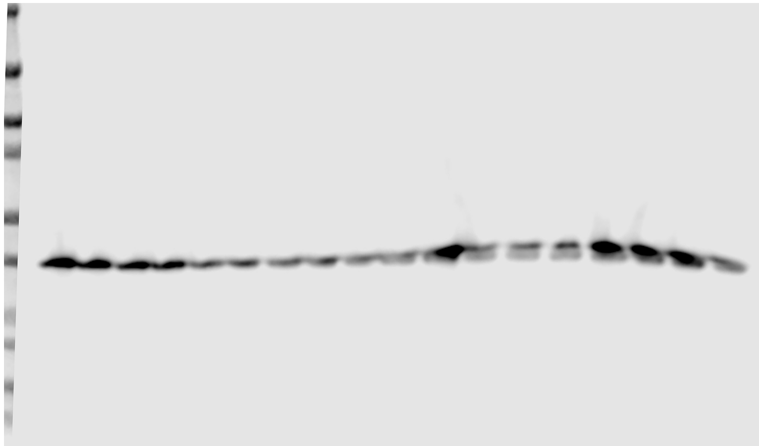

P-PKA Substrates

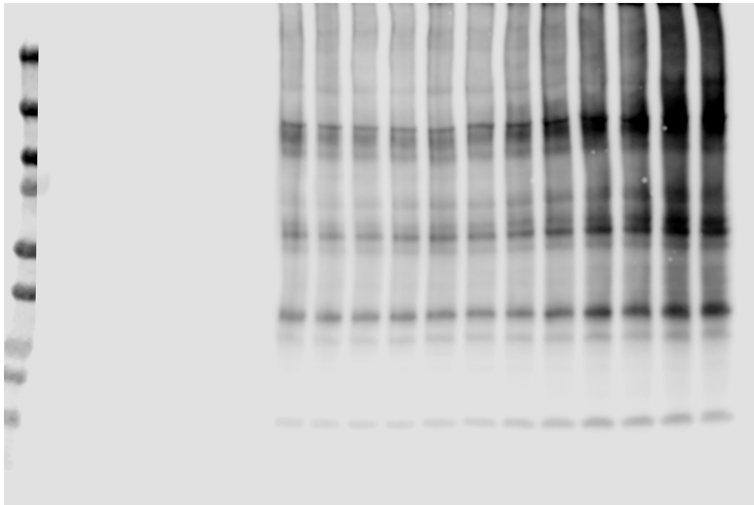

Supplemental Figure 7E

UCP1

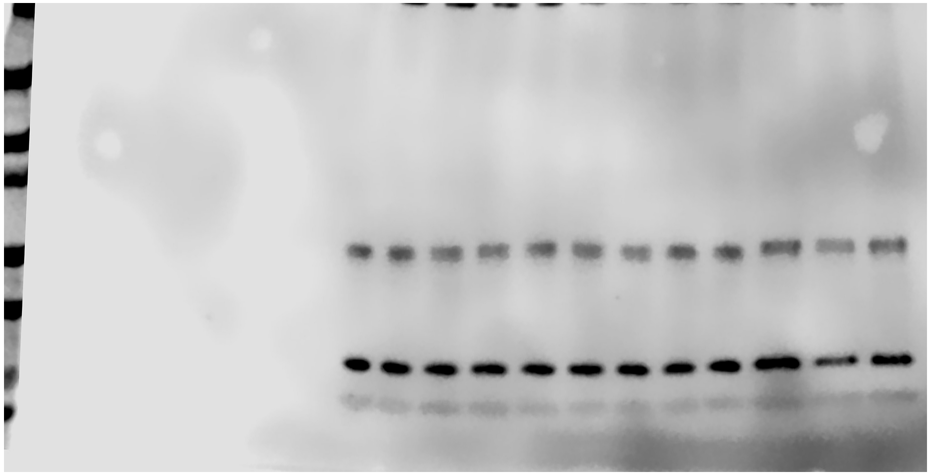

P-PKA Substrates

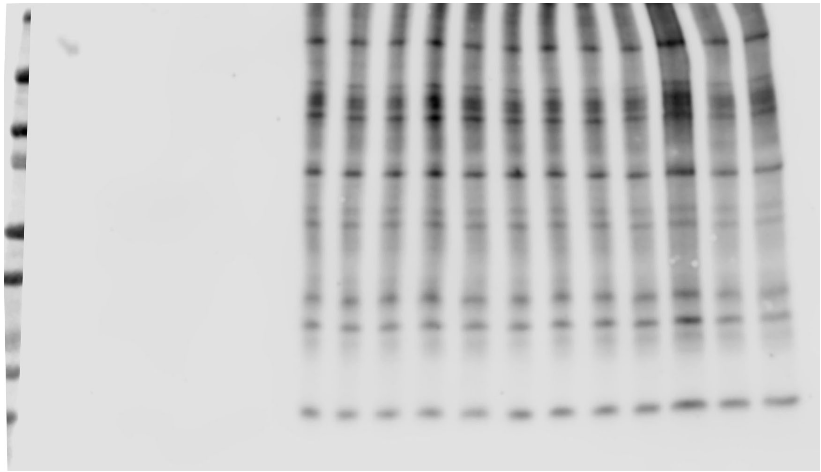

GAPDH

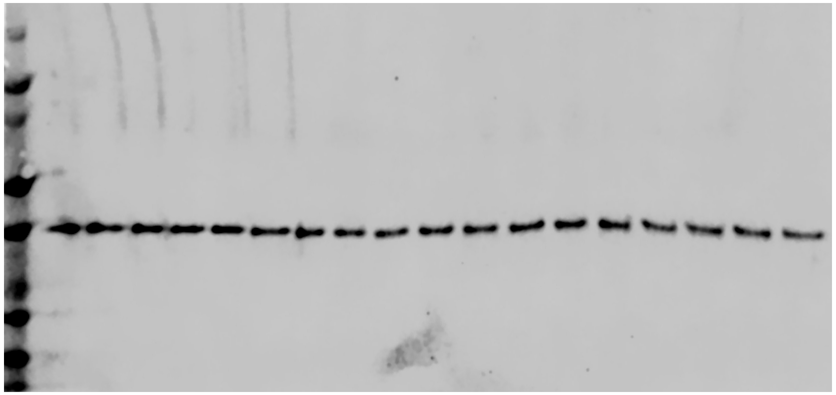

Supplemental Figure 7G

P-PKA Substrates

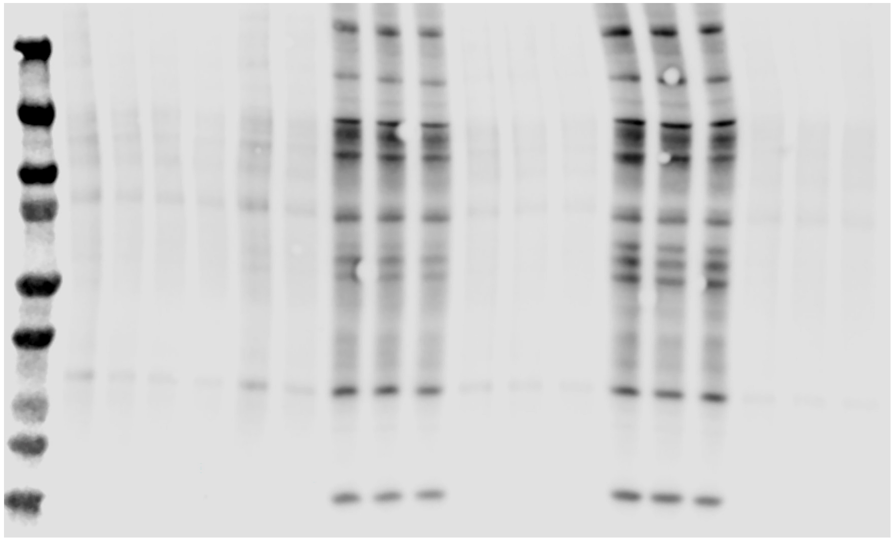

GAPDH

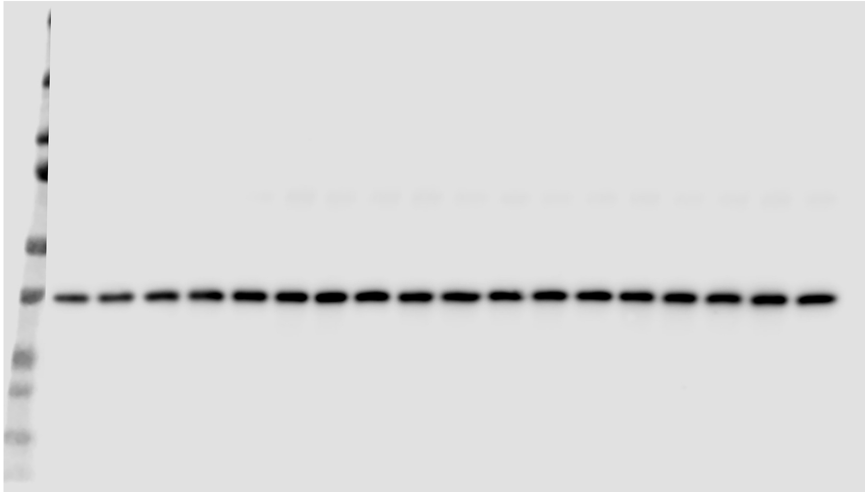

Supplemental Figure 7H

P-PKA Substrates

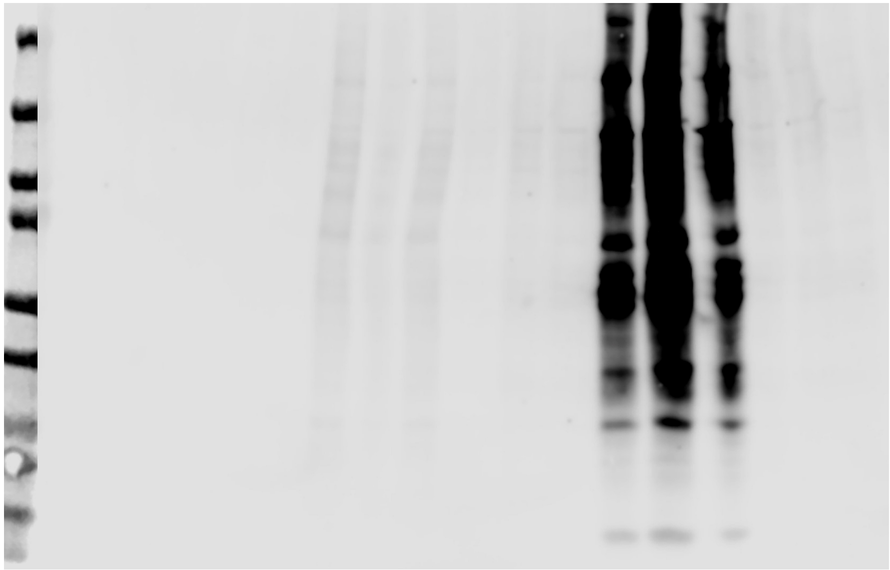

GAPDH

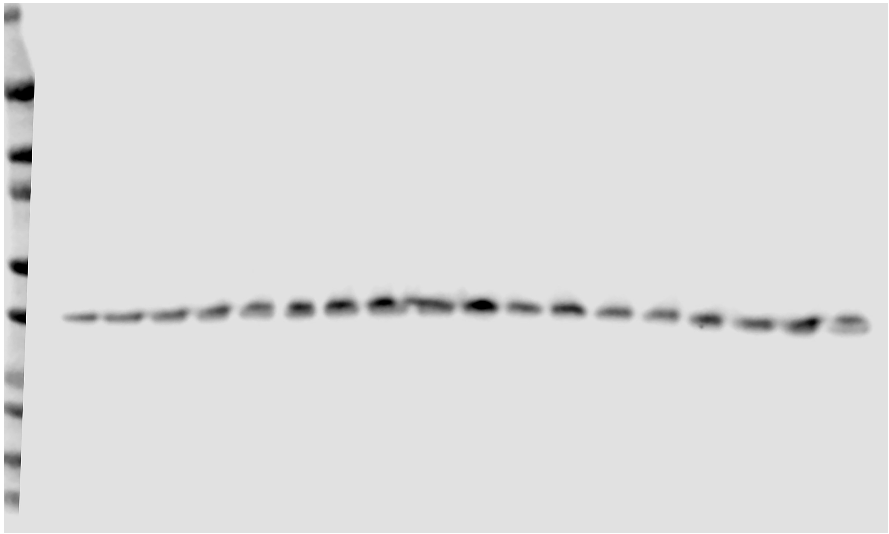

Supplemental Figure 8

β_3-AR

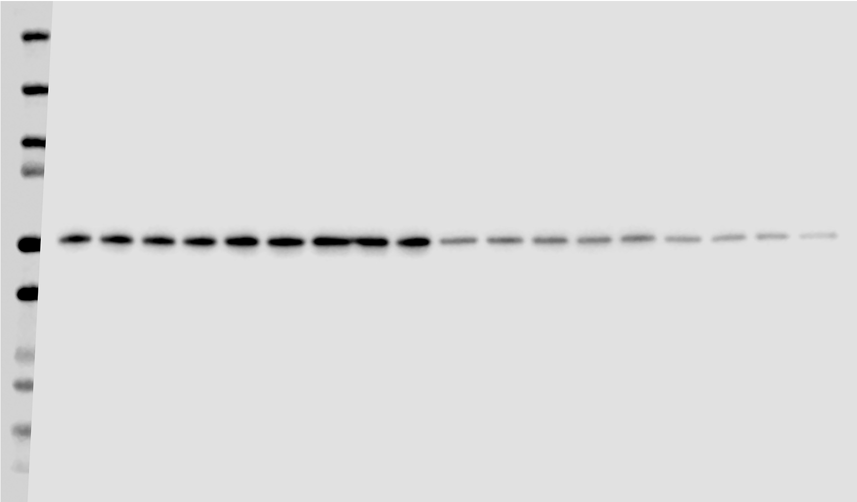

UCP1

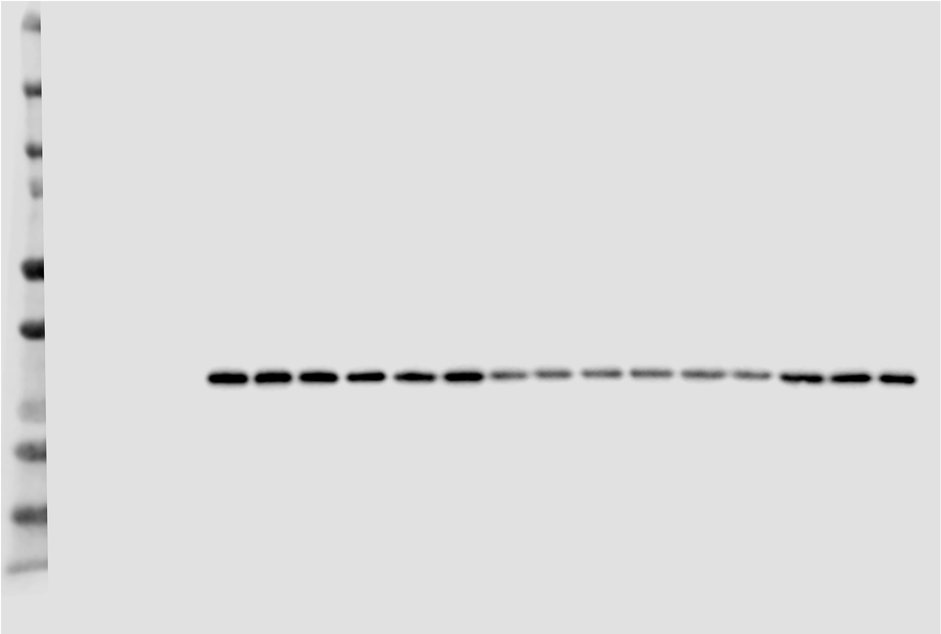

GAPDH

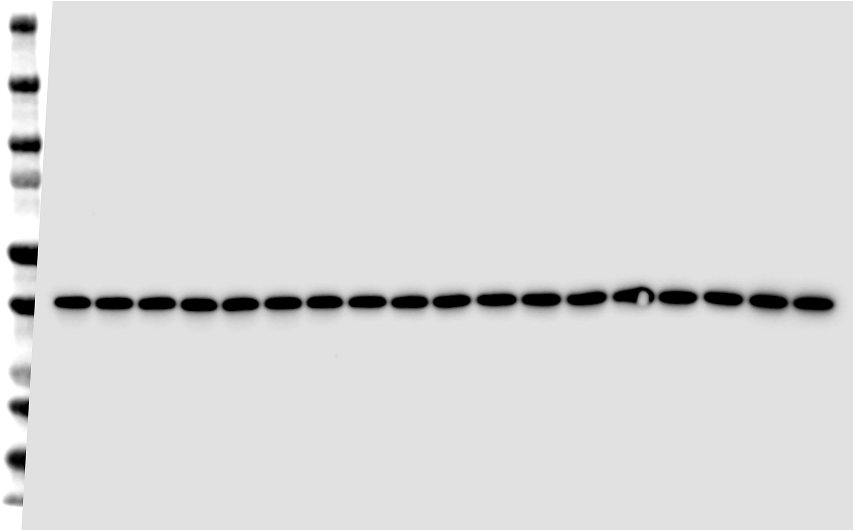

P-PKA Substrates

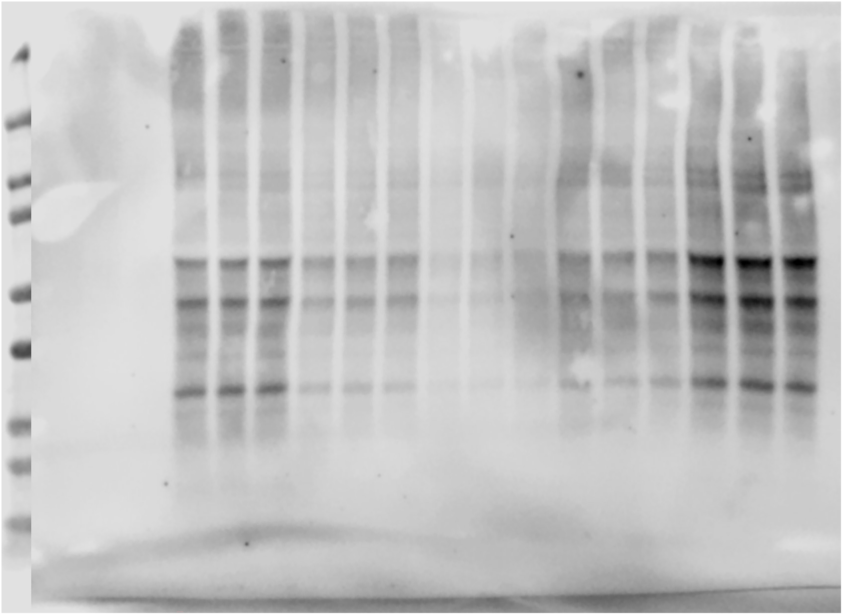

Supplemental Figure 9

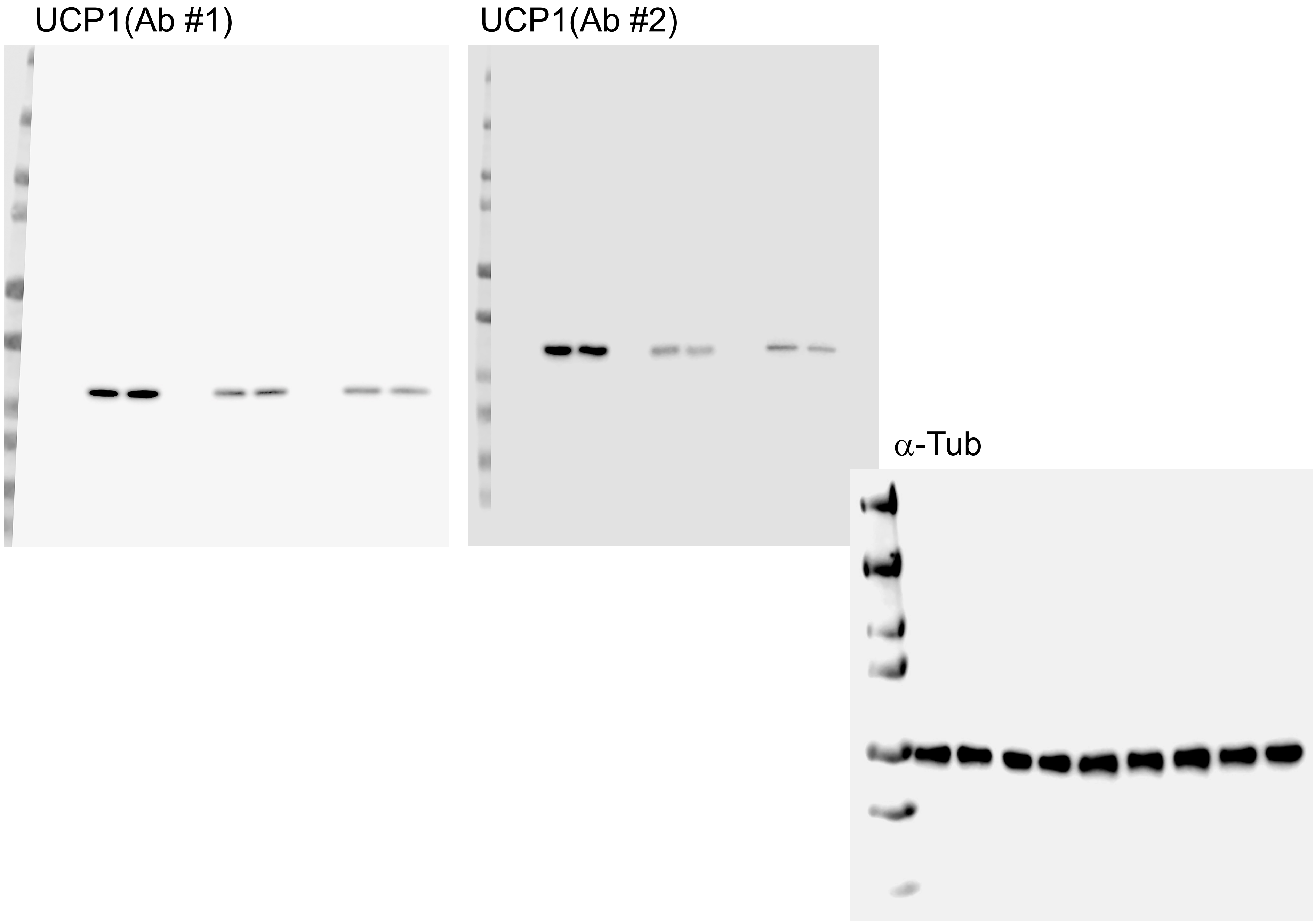

Supplement: Supplemental data [file jci-133-168192-s011.pdf]
